# Insights into the Mechanism Underlying the Alkane Dehydrogenation Capability of *Rhodococcus* sp. KSM-B-3M

**DOI:** 10.3390/microorganisms14061252

**Published:** 2026-06-02

**Authors:** Nurit Edri, Keren Buhnik-Rosenblau, Anthony Cohen, Shlomit Hakim, Fabian Glaser, Guy Horev, Jeffrey Bruffaerts, Ilan Marek, Yechezkel Kashi

**Affiliations:** 1 Department of Biotechnology and Food Engineering, Technion—Israel Institute of Technology, Haifa 3200003, Israel; nurit.edri@biu.ac.il (N.E.); buhnik@technion.ac.il (K.B.-R.); shlomithakim29@gmail.com (S.H.); 2 The Resnick Sustainability Center for Catalysis, Schulich Faculty of Chemistry, Technion—Israel Institute of Technology, Haifa 3200008, Israel; anthonycohen4@gmail.com (A.C.); jeffrey.bruffaerts@gmail.com (J.B.); chilanm@technion.ac.il (I.M.); 3 The Lorry I. Lokey Center for Life Sciences and Engineering, Technion—Israel Institute of Technology, Haifa 3200003, Israel; fglaser@technion.ac.il (F.G.); guyh@migal.org.il (G.H.)

**Keywords:** alkane dehydrogenation, alkenes, *Rhodococcus* sp. KSM-B-3M, Acyl-CoA desaturase, fatty acid degradation

## Abstract

Alkanes are saturated hydrocarbons that serve as available and cost-effective feedstock for producing alkenes, key intermediates in numerous industrial processes. A mutant bacterial strain, *Rhodococcus* sp. KSM-B-3M, was previously reported to efficiently convert alkanes into alkenes and was later utilized by us to selectively transform linear alkanes into a variety of alkyl derivatives through a two-step process. Here, we explored the biological mechanisms underlying the unique biotransformation capability of strain KSM-B-3M by integrating genomics, transcriptomics, proteomics, and 3D-structural modelling. Strain KSM-B-3M demonstrated downregulation of the fatty acid degradation pathway, lacking the pR8L1 megaplasmid that carries multiple fatty acid degradation genes, accompanied by a parallel high expression of the acyl CoA-desaturase gene. Partial curing of the pR8L1 plasmid from a wild-type (WT) strain conferred the ability to dehydrogenate *n*-hexadecane to *cis*-hexadecene. Overexpression of the acyl-CoA desaturase gene similarly induced *cis*-hexadecene formation in the WT strain, acting cumulatively with fatty acid degradation downregulation. Acyl CoA-desaturase 3-D modeling suggested that the enzyme directly dehydrogenates *n*-hexadecane to form *cis*-hexadecene, supporting its direct role in this unique biotransformation. These findings advance our understanding of the mechanism behind this biotransformation, which holds promise for sustainable and cost-effective production of alkyl derivatives.

## 1. Background

Alkanes are saturated hydrocarbons, varying in size and structure [1]. They are a primary component of natural gas and crude oil [2] and considered a promising energy storage source along with other materials [3,4]. Due to their abundance, alkanes are readily available and cost-effective raw materials for producing alkenes, unsaturated hydrocarbons heavily used as key intermediates in the pharmaceutical industry [5]. Alkanes, being chemically inert, are typically functionalized and converted to alkenes through low-yield chemical reactions, requiring high temperatures necessary to break the energetically akin C−H bonds [6]. Exceptionally, a unique and remarkable alkane conversion capability has been observed in a mutant strain, KSM-B-3M, of the *Rhodococcus* genus [7,8]. Rhodococci, known for their ability to thrive in harsh environments like oil-contaminated soils [9], show promise for bioremediation, biocatalysis, and biotransformation due to their metabolic flexibility [10]. This includes the capability to uptake alkanes and degrade them through various oxidation pathways [1,2,11,12], such as the beta oxidation pathway, resulting in the formation of acetyl-CoA. The mutant strain *Rhodococcus* sp. KSM-B-3M stands out for its unique ability to metabolize alkanes into alkenes with identical chain lengths. Alkenes are produced in a regioselective manner, yielding *cis*-7- and *cis*-8-hexadecene [7,8] from *n*-hexadecane. Leveraging this distinctive capability of *Rhodococcus* sp. KSM-B-3M, a two-step strategy involving dehydrogenation and remote hydrofunctionalization, has been demonstrated as a unified and versatile approach to selectively convert linear alkanes into a large array of valuable functionalized derivatives [8]. This biological system offers advantages over traditional chemical synthesis methods [8,13], minimizing side reactions and achieving a synthetically useful yield of 61% [8]. Nonetheless, the mechanism behind the alkane dehydrogenation ability of *Rhodococcus* sp. KSM-B-3M remains largely unexplored. The current research aims to uncover the metabolic pathways and enzymatic mechanisms responsible for this efficient process by integrating genomic, transcriptomic, and proteomic analyses in comparison with a non-dehydrogenating reference strain. Modeling was employed as a predictive strategy [14,15,16], and genetic engineering approaches were further employed to validate the involvement of downregulation of the fatty acid degradation pathway and overexpression of acyl-CoA desaturase in alkane dehydrogenation.

These findings hold potential for enhancing alkane conversion efficiency in the industrial production of alkenes using sustainable microbial systems.

## 2. Materials and Methods

### 2.1. Culture Growth and Dehydrogenation Conditions

Two *Rhodococcus* strains, *Rhodococcus* sp. KSM-B-3M [7] and *Rhodococcus* sp. 008, were used for this study. Starter cultures were grown for 20–24 h in Nutrient Broth (NB; Cat. No. 70149, Sigma-Aldrich. St. Louis, MO, USA) at 30 °C under agitation of 180 rpm. To set up a dehydrogenation reaction, we followed our previously published protocol [6]. Briefly, starter cultures were sub-cultured by transferring 1 mL to fresh NB (200 mL, in a 500 mL Erlenmeyer) for an additional 20–24 h incubation step at 30 °C, 180 rpm. Bacterial cells were harvested (4800 g, 10 min) and washed with 5 mL sterile saline solution (0.9% *w*/*v* sodium chloride) to remove medium leftovers. One gram of wet bacterial culture was dissolved in 57.5 mL of freshly prepared sterile medium A (0.46 M KH_2_PO_4_, 0.32 M K_2_HPO_4_, 21.3 mM monosodium glutamate (MSG), 0.3 mM thiamine hydrochloride, and 0.4 mM magnesium sulphate heptahydrate; pH 6.4) supplemented with 8.5 mmol of the neat substrate (alkane or fatty acid) in sterile 250 mL Erlenmeyer flasks bearing breathable corks. Flasks were incubated (180 rpm, 30 °C) over the course of seven days, and alkene formation was tested by gas chromatograph (GC) analysis. Samples were prepared for GC analysis by mixing 0.5 mL of the cultures with 1 mL of absolute ethanol, followed by organic phase separation with ethyl acetate (EtOAc) and purification through a short pad of silica gel. The dehydrogenation (conversion) ratio was defined as the product abundance relative to the substrate abundance. The location of the formed double bond was identified through oxidative cleavage of the compound, followed by GC analysis and chemical correlations with known compounds [17].

### 2.2. RNA Extraction

Prior to the RNA extraction procedure, cells were recovered through the following protocols: 

Recovery of Rhodococcus sp. KSM B-3M or sp. 008 cells from medium A supplemented with alkanes: 15 mL of each culture was centrifuged (400 g, 1 min) to separate the aqueous and organic phases. The organic phase (0.5–1 mL fractions) was transferred to a new tube and centrifuged again (9600 g, 1 min), and the intermediate phase containing bacterial cells was dissolved in two volumes of RNAprotect Bacteria Reagent (Cat No. 76506, Qiagen, Hilden, Germany). Samples were mixed using constant vortex for 5 min, cells were pelleted by centrifugation (9600 g, 2 min), and supernatant was removed. All centrifugation steps were conducted at room temperature (RT).

Recovery of Rhodococcus sp. KSM B-3M or sp. 008 cells from medium A supplemented with fatty acids: 2 mL of each culture were mixed with two volumes of RNAprotect Bacteria Reagent (Cat No. 76506, Qiagen, Hilden, Germany) and incubated for 5 min at RT. Cells were pelleted by centrifugation (4700 rpm, 10 min, RT) and supernatant was removed.

RNA extraction: Cell pellets were suspended in 100 µL lysozyme solution (15 mg/mL in Tris-EDTA buffer [TE]; Cat No. L6876, Sigma Aldrich, St. Louis, MO, USA), mixed with 20 µL proteinase k solution (Cat No. 19131, Qiagen, Hilden, Germany). The suspension was mixed by vortex for 10 s every 2 min along a 10 min course. 700 µL buffer RLT (RNeasy mini kit, Cat No. 74104, Qiagen, Hilden, Germany) supplied with 0.1% (*v*/*v*) 2-mercaptoethanol was added, followed by vortexing for 10 s and a successive mechanical disruption step of constant vortex in the presence of 50 mg acid-washed glass beads (Cat No. G8772, Sigma Aldrich, St. Louis, MO, USA) for 5 min. Samples were centrifuged (10 s, maximum speed) and 760 µL of the supernatant was transferred to a new tube. 590 µL ethanol 80% were added to the supernatant and shaken vigorously. RNA was further isolated using the Rneasy mini kit (Cat No. 74104, Qiagen, Hilden, Germany) according to the manufacturer’s instructions. To remove DNA, RNA samples were incubated for 1 h with 5 µL DNase I stock solution, 10 µL of RDD buffer (Cat No. 79254, Qiagen, Hilden, Germany), and RNAse-free water to a 100 µL adjusted volume. RNA cleanup step (Rneasy mini kit, Cat No. 74104, Qiagen, Hilden, Germany) was further performed according to the manufacturer’s instructions. DNase treatment was performed twice to fully eliminate DNA contamination, verified by the absence of a PCR amplification product targeting the 16S rDNA sequences (using primers 16S and 16R, Supplementary Table S1), with the extracted RNA samples as a template. RNA quality and quantity were determined using Agilent RNA ScreenTape^®^ (Agilent, Santa Clara, CA, USA) and Qubit RNA High Sensitivity kit (Thermo Fischer Scientific, Waltham, MA, USA), respectively.

### 2.3. PCR

Each PCR mixture contained 1xBio-ReadyMix (Cat No. 959758026500, Bio-lab, Jerusalem, Israel), 0.4 μM forward and reverse primers, and 2 μL template (purified or crude DNA for colony PCR, prepared by suspending 5 µL O.N culture in 25 µL of DDW and incubating at 98 °C for 10 min) in a total volume of 25 μL. The reactions were carried out in a Veriti 96-well thermal cycler (Applied Biosystems, Waltham, MA, USA) as follows: 95 °C for 5 min; 30 cycles of 15 s at 95 °C, 15 s at the annealing temperature (Supplementary Table S1), and 30 s for every 1 kb at 72 °C; 7 min at 72 °C, and cooling to 12 °C. PCR products were verified by gel (1.2%) electrophoresis and observed by UV fluorescence.

### 2.4. Quantitative PCR (qPCR)

Reverse transcription was performed using a High Capacity cDNA Reverse Transcription Kit (Cat No. 4368814, Thermo Fisher Scientific, Waltham, MA, USA), according to the manufacturer’s instructions. qPCR reactions contained 1X Fast SYBR™ Green Master Mix (Cat. No. AB-4385612, Thermo Fisher Scientific, Waltham, MA, USA), 2 ng template (the synthesized cDNA or DNA samples—purified or crude DNA prepared by suspending 5 µL O.N culture in 25 µL of DDW and incubating at 98 °C for 10 min), 25 μM (0.3 μL each) of the appropriate forward and reverse PCR primers (Supplementary Table S1) and DDW to a total volume of 10 µL. The qPCR reactions were conducted in a Step One Plus Real-Time PCR System (Applied Biosystems) as follows: 95 °C for 20 s and 40 cycles of 95 °C for 3 s followed by 60 °C for 30 s. Post-amplification melting was performed using increasing temperature from 60 °C to 95 °C at a rate of 0.3 °C every second, with a final incubation for 15 s at 95 °C. Results were analyzed using the StepOne Software v2.3 (Applied Biosystems). Gene expression was calculated using the comparative Ct method (2^−ΔCt^) [18] or as relative expression (RQ) using the ΔΔCt method with 23s rDNA or 16s rDNA as the reference gene, as described before [19], where ΔΔCt = ΔCt (transformant clone) − ΔCt (original clone); ΔCt = Ct (gene of interest) − Ct (reference gene) and the fold gene expression = 2^−(ΔΔCt)^.

### 2.5. RNAseq

For the *n*-hexadecane/*n*-dodecane RNAseq experiment, sequencing libraries were prepared starting with total RNA using Illumina ScriptSeq Complete Kit (Bacteria) (Illumina, San Diego, CA, USA) according to manufacturer instructions. Sequencing was performed on Illumina HiSeq 2500 using one 50 SR high-output lane. For the *n*-hexadecane/palmitic acid RNAseq experiment, sequencing libraries were prepared using the Illumina Stranded Total RNA Prep Ligation RiboZero (Illumina, cat no. 20040525, San Diego, CA, USA) according to the manufacturer’s protocol. Libraries were sequenced on the Illumina NextSeq 2000 instrument with 100 bps single-read. RNAseq was carried out in biological triplicates, normalization and differential expression analyses were conducted using RUVseq and DESeq2 R package version 1.10.0 [20]. Principal component analysis (PCA) and volcano plots were generated using DESeq2 and tidyverse packages in the R software (v4.1.1; R Core Team 2021) environment, respectively.

### 2.6. DNA Extraction

A loop full of overnight (O.N) bacterial cultures was suspended in 1 mL of 1xSSC solution (3 M NaCl, 0.3 M Sodium citrate; pH 7) and mixed by vortex. Cells were harvested by centrifugation (1 min, maximum speed), resuspended in 100 µL lysozyme solution (0.4% *w*/*v* lysozyme [Cat No. L6876, Sigma Aldrich, St. Louis, MO, USA], 1 mM sodium phosphate in 20% sucrose), and incubated for 1 h at 37 °C. 200 µL TE, 100 µL sarkosyl solution (0.5% Sodium lauryl sarcosinate in TE buffer), and 20 µL proteinase K (Cat No. 19131, Qiagen, Hilden, Germany) were added and mixed by gentle vortex. Following an O.N. incubation at 50 °C, 500 µL EZ-DNA (cat no. 20-600-50, Biological Industries, Beit Haemek, Israel) was added, and an additional incubation step was carried out at 60 °C for 1 h. 1 mL of absolute ethanol was then added, the tubes were inverted to mix the suspension, and centrifuged (5000 g, 4 min). The pellet was washed with 95% ethanol, dried for 30 min at 37 °C, resuspended in 500 µL TE supplemented with 6 µL RNase A solution (10 mg/mL RNase A [Cat. No. R4875, Sigma Aldrich, St. Louis, MO, USA], 0.01 M sodium acetate, 0.1 volume of 1 M Tris-HCl pH 7.4), and incubated at 37 °C for 1 h. An equal volume of phenol was added, the tubes were mixed by a gentle vortex, and then centrifuged (5 min, maximum speed). Upper phase was transferred to a new Eppendorf tube, mixed with an equal volume of chloroform: isoamyl alcohol (1:24), and centrifuged again (5 min, maximum speed). To pellet the DNA, the upper phase was mixed with 1/10 volume 3 M sodium acetate and 2 volumes of absolute ethanol, incubated for 15 min at −80 °C, and centrifuged (5 min, maximum speed). The dried pellet was resuspended in 30 µL 0.1xTE.

### 2.7. Oxford Nanopore DNA Sequencing

DNA concentration was measured using the Qubit Flex fluorometer (Invitrogen) with the Equalbit dsDNA HS Assay Kit (Vazyme, cat. no. EQ121, Nanjing, China). Fragment size analysis was performed using the TapeStation 4200 system with the Genomic DNA ScreenTape kit (Agilent, cat. no. 5067-5365, Santa Clara, CA, USA), exhibiting high molecular-weight genomic DNA. Genomic DNA libraries were prepared in parallel using the Native Barcoding Kit V14 (Oxford Nanopore, cat. no. SQK-NBD114.24), following the manufacturer’s protocol. For library construction, 400 ng and 1000 ng DNA were taken and pooled after barcoding for subsequent ONT adaptor ligation. Quality control of the pooled Nanopore libraries included concentration measurement using the Qubit Flex (Thermo Fisher Scientific) and Equalbit dsDNA HS Assay Kit (Vazyme, cat. no. EQ121, Nanjing, China), and fragment size assessment using the TapeStation 4200 system with the Genomic DNA ScreenTape kit (Agilent, cat. no. 5067-5365, Santa Clara, CA, USA). The sequencing data were generated on the Oxford Nanopore MinION device (MinKNOW software v.25.03.9), using R10.4.1 flow cell (Oxford Nanopore, cat no. FLO-MIN114, Oxford Science Park, United Kingdom). Raw POD5 files were base called and demultiplexed by Dorado Software (v.0.8.3), using the ‘sup’ basecalling model (dna_r10.4.1_e8.2_400bps_sup@v5.0.0). Official NCBI RefSeq assembly GCF_001682295.1 was downloaded, including: Chromosome (CP015206.1), plasmid pR8L1 (CP015203.1), plasmid pR8C1 (CP015204.1), and plasmid pR8C2 (CP015205.1). Reference sequences were concatenated into a unified reference database. Large deletions and cured (lost) genomic regions were identified using a coverage-based approach based on depth calculation using samtools depth -aa and calculating log_2_ ratios, where regions with log_2_ratio < −3 were flagged as deleted. Small variants were screened using the Epi2Me wf-bacterial-genomes pipeline. bcftools isec was used to remove systematic sequencing errors.

### 2.8. Phylogenetic Analysis and Genome Comparison of Rhodococcus Strains

To identify the genetically closest *Rhodococcus* strain to KSM B-3M, whole genome sequencing was performed, and the assembly of KSM B-3M was generated using SPAdes [21]. RealPhy [22] was used to reconstruct the phylogenetic tree that includes unassembled reads of KSM B-3M, the assembly of KSM B-3M, 13 published genomes of *Rhodococcus,* and two genomes of *Nocardia farcinica* strains that served as an outgroup. Mapping of the reads was performed with bowtie2 [23]. PhyML was used to build the tree [24]. The R package ggtree [25] was used to generate a phylogram visualization of the tree. Comparison of KSM B-3M and sp. 008 chromosomes and mega-plasmids was carried out using the NCBI Blast algorithm [26].

### 2.9. Proteolysis and Mass Spectrometry Analysis

Cell pellet samples were dissolved in 10 mM DTT, 100 mM Tris, and 5% SDS. Glass beads were added, and the samples were vortexed for 30 min in a bead beater. The supernatant was transferred to a clean tube and sonicated twice (5 min, 10–10, 90%), followed by boiling at 95 °C for 10 min. The samples were precipitated in 80% acetone in water and washed 3 times with 80% acetone in water. The protein pellets were dissolved in 9 M urea and 400 mM ammonium bicarbonate, then reduced with 10 mM DTT (60 °C for 30 min), carboxymethylated with 35 mM iodoacetamide (room temperature for 30 min in the dark), and digested in 2 M urea, 66 mM ammonium bicarbonate with modified trypsin (Promega), at a 1:50 enzyme-to-substrate ratio, overnight at 37 °C. An additional second trypsinization was performed for 4 h in 1 M urea.

The resulting tryptic peptides were desalted using C18 stage-tips (homemade from 3 M Empore disks of either C18 or SCX material), dried, re-suspended in 0.1% TFA, and cleaned on SCX stage-tips (homemade). The peptides were resolved by reverse-phase chromatography on 0.075 × 180-mm fused silica capillaries (J&W) packed with Reprosil reversed-phase material (Dr Maisch GmbH, Germany, Ammerbuch, Germany). The peptides were resolved by reverse-phase chromatography on 0.075 X 180 mm fused silica capillaries (J&W) packed with Reprosil reversed-phase material (Dr Maisch GmbH, Germany, Ammerbuch, Germany). The peptides were eluted with a linear gradient of 5 to 28% acetonitrile with 0.1% formic acid for 180 min, 15 min with a gradient of 28 to 95%, and 25 min at 95% acetonitrile with 0.1% formic acid in water at flow rates of 0.15 μL/min. Mass spectrometry was performed by a Q-Exactive Plus mass spectrometer (Thermo) in a positive mode using a repetitively full MS scan followed by collision-induced dissociation (HCD) of the 10 most dominant ions selected from the first MS scan.

The mass spectrometry data were analyzed using Proteome Discoverer 1.4 software with Sequest (Thermo) algorithm against the Rhodococcus-erythropolis proteome from the Uniprot database, with 1% FDR. Semi-quantitation was performed by calculating the peak area of each peptide based on its extracted ion currents (XICs), and the area of the protein is the average of the three most intense peptides from each protein.

Enrichment analysis was carried out using DAVID [27,28].

### 2.10. Plasmid Curing

0.1 OD_600_ of *Rhodococcus* sp. 008 O.N culture was grown in LB medium containing 5, 10, or 15 µg/mL acridine orange (cat. No. A6014, Sigma Aldrich, St. Louis, MO, USA) at 30 °C for 24 h under orbital agitation. Over 1000 clones isolated from the curing procedure were recovered on Luria agar (LA) for 24 h at 30 °C, resuspended in 50% cryogenic preservation medium (50% *v*/*v* glycerol, 50% *v*/*v* LB), and stored at −80 °C until further analysis. Clones were screened for cured pR8L1 plasmid by qPCR targeting the acyl CoA ligase gene (WP_003942422.1; primers Ligase_RT-F and Ligase_RT-R, Supplementary Table S1) located on the pR8L1 plasmid, at an annealing and extension temperature of 64 °C. DNA extracted from *Rhodococcus* sp. KSM B-3M or sp. 008 served as negative and positive controls, respectively. Clones with Ct value higher than 20 were further evaluated by multiplex PCR targeting both acyl CoA ligase gene and the 16S rDNA as an internal control (using primers Ligase1-F and Ligase1-R, and primers 16S-F and 16S-R, respectively; Supplementary Table S1). To roughly demonstrate the deletion boundaries of cured clones, additional PCR reactions targeting various loci spanning along plasmid pR8L1 were carried out (primers PR8L1-1-7, Supplementary Table S1). This was carried out alongside a further assessment of other genetic loci distributed along the additional plasmids pR8C1 and pR8C2 (primers pR8C1_1-3 and pR8C2_1-3, Supplementary Table S1), in order to confirm that large DNA fragments were not cured from these plasmids.

### 2.11. Rhodococcus Acyl CoA Desaturase 3D Structure

Models of the *Rhodococcus* acyl-CoA desaturase complex with the hexadecane and two Zn ions docked were obtained by the RoseTTAFold All-Atom tool. RoseTTAFold All-Atom is a next-generation protein structure prediction and design tool, which demonstrates superior performance on protein-ligand structure prediction relative to other tools, even in the absence of an input experimental structure [29]. To explore flexibility, we used ColabFold [30] to produce multiple AlphaFold models using a deep sampling protocol from [15], which shows that AlphaFold2 can directly predict the relative populations of different protein conformations by subsampling multiple sequence alignments. We run AlphaFold2 with the following parameters: --num-seeds 32 --max-msa 64:128 dropout, producing a total of 160 models with 32 different seeds. This large set of models provides a higher structural variance by subsampling the original full MSA. This is in accordance with previous works that have shown that a significant reduction in the values of max_seq and extra_seq parameters from their default values (512:2048) achieves ensemble prediction for a series of model systems [16]. Molecular graphics and analyses performed with UCSF ChimeraX [31], developed by the Resource for Biocomputing, Visualization, and Informatics at the University of California, San Francisco, with support from National Institutes of Health R01-GM129325 and the Office of Cyber Infrastructure and Computational Biology, National Institute of Allergy and Infectious Diseases. Molecular lipophilicity potential (MLP) [32] maps are computed within ChimeraX with the command mlp, calculated for protein atoms only, and mapped into the molecular surface of the molecule. Structural Alignments within ChimeraX are performed with the MatchMaker tool [33], using a single atom per residue (CA).

### 2.12. Acyl-CoA Desaturase Overexpression

Overexpression of acyl-CoA desaturase (WP_003945297.1) or the entire putative operon (WP_003945295.1, WP_003945297.1, WP_050656813.1) was carried out in the pDD120 vector, previously used for constitutive overexpression in *Rhodococcus opacus* PD630 [34]. The acyl-CoA desaturase gene or the three putative operon genes were amplified from the genomic DNA of *Rhodococcus* sp. KSM-B-3M (using primers desaturase_NdeI_F and desaturase_ApaI_R or Operon_NdeI_F and Operon_ApaI_R, respectively; Supplementary Table S1). The purified PCR product was ligated into the pDD120 backbone digested in its NdeI (Cat. No. R0111S, New England Biolabs, Ipswich, MA, USA) and ApaI (Cat. No. R0114S, New England Biolabs, Ipswich, MA, USA) restriction sites, to produce the acyl-CoA desaturase overexpressing plasmid pD1, or the entire operon overexpressing plasmid pO1. Plasmids were introduced by electroporation into *Rhodococcus* sp. 008 or clones *Rhodococcus* sp. pKO3-5 cured in their pR8L1 plasmid, based on competent cells preparation and electroporation protocols previously described by Van Der Geize et al. [35]. Briefly, half loop of O.N *Rhodococcus* cultures grown on NB plates was suspended in 3 mL LBSG medium (1% Bacto Tryptone, 0.5% Bacto Yeast extract, 0.5% NaCl, 10% Sucrose and 3% Glycine) and grown for 24 h at 30 °C, 180 rpm. 2 mL portions were transferred to 500 mL Erlenmeyer flask containing 100 mL LBSG medium, grown until late-exponential phase (OD_600_ 2–3) and cooled on ice. Cells were harvested by centrifugation (10 min, 4000 g, 4 °C) and washed twice with cold distilled water. The pellets were resuspended in 1 mL of cold 30% (*v*/*v*) polyethylene glycol 1450. 100 µL aliquots were frozen under liquid nitrogen and kept at −80 °C until use. For each transformation, a single competent cell aliquot was thawed on ice, supplemented with 1 µg of pD1 or pO1 plasmids, and transferred to 2 mm precooled gapped cuvettes (Cat No. EP-102, cell projects). Cells were kept on ice for 10 min and then electroporated using a single 3.0 kV pulse in a MicroPulser™ electroporation apparatus (Bio-Rad). 1 mL LB was added immediately after the electroporation, followed by a 4.5 h incubation step at 30 °C under moderate agitation. Cells were grown on LA plates supplemented with 90 µg/mL kanamycin to obtain transformants after 2–3 incubation days at 30 °C. Verification of transformants was carried out by amplifying the acyl-CoA desaturase or the entire operon flanking regions on the plasmid backbone to obtain a 1338 bp or 2682bp product, respectively (using primers pDD120_seq-F and pDD120_seq-R; Supplementary Table S1).

### 2.13. In Vitro Desaturase Assay

20 µg recombinant Acyl-CoA desaturase 1 (Scd1; MBS1201181, MyBioSource, San Diego, CA, USA) or custom-made KSM-B-3M *Rhodococcal* Acyl-CoA desaturase produced via in vitro *E. coli* expression system (MyBioSource) and 2.9 µL *n*-hexadecane were suspended in 200 µL of desaturase assay buffer (0.33 M sucrose (7654, Merck, Darmstadt, Germany), 1 mM Phenylmethylsulfonyl fluoride (P7626, Sigma Aldrich, St. Louis, MO, USA), 0.18% bovine serum albumin (A7906, Sigma Aldrich, St. Louis, MO, USA), 0.5% NADPH (10107824001, Roche Diagnostics GmbH, Mannheim, Germany) in 0.1 M Potassium phosphate buffer pH 7.2; [36]) and incubated at 30 °C for 48 h. Formation of *cis*-hexadecene was tested by GC analysis as described above.

### 2.14. Statistical Analysis

Differences in conversion ratios among groups were determined using the Wilcoxon rank sum test or the Kruskal–Wallis rank sum test followed by the DUNN test, using the rstatix [37] package in the R Software (v4.1.1; R Core Team 2021) [38].

## 3. Results

The capability of *Rhodococcus* sp. KSM-B-3M to convert alkanes into their corresponding alkenes has been previously described by us and others [7,8], yet the biological mechanism underlying this activity remained unclear. To investigate this phenomenon, our initial objective was to identify a closely related strain lacking conversion ability to serve as a reference in the study. Since we did not have access to the parental strain of *Rhodococcus* sp. KSM-B-3M (*Rhodococcus* sp. KSM-B [7]; which lacked detectable alkane dehydrogenation ability), we compared the genome of strain KSM-B-3M with other rhodococci available genomes to find the most closely related strain. Phylogenetic analysis identified *Rhodococcus* sp. 008 as the closest known genetically related strain to KSM-B-3M (Supplementary Figure S1). Strain sp. 008, which showed no detectable alkane dehydrogenation ability, was designated as the reference strain for this study.

**Figure 1:**
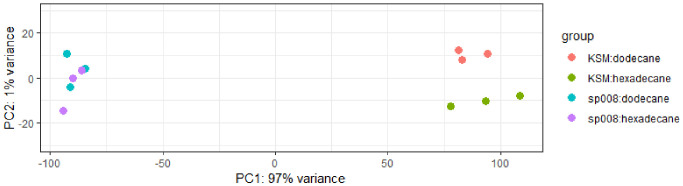
The transcriptomic profiles of strains KSM-B-3M and sp. 008 grown on *n*-hexadecane and *n*-dodecane. The profiles are demonstrated using principal component analysis. Pink, strain KSM-B-3M grown on *n*-dodecane; green, strain KSM-B-3M grown on *n*-hexadecane; blue, strain sp. 008 grown on *n*-dodecane; purple, strain sp. 008 grown on *n*-hexadecane. 97% of the variation is explained by PC1, which corresponds to differences between strains.

To pinpoint the genes and pathways responsible for alkane dehydrogenation in strain KSM-B-3M, transcriptomic analysis was conducted. We compared the transcriptomes of the two strains (*Rhodococcus* sp. KSM-B-3M and sp. 008) grown on *n*-hexadecane and *n*-dodecane associated with high (~50%) and low (<5%) dehydrogenation ratios by strain KSM-B-3M, respectively. The variation in gene expression profiles was primarily strain-dependent, with 97% of the variation in gene expression linked to differences between strains (PC1; Figure 1). Only 1% of the observed variation, specific to strain KSM-B-3M, was associated with differences in substrate (PC2; Figure 1).

**Figure 2:**
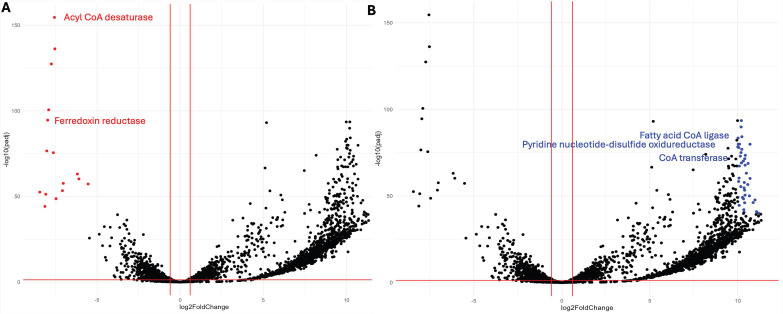
Differential gene expression between strains KSM-B-3M and sp. 008 grown on *n*-hexadecane. Volcano plots representing the significance of each gene expression vs. its expression differential. (**A**). Genes in red are excessive in strain KSM-B-3M by at least 2^5^ fold change. (**B**). Genes in blue are excessive in strain sp. 008 by at least 2^10^ fold change. Vertical red lines mark -0.6, 0.6 boundaries on the x axis.

Differential gene expression analysis focusing on *n*-hexadecane revealed 1331 genes that were differently expressed between the two strains, with at least a ten-fold excess (adjusted *p*-value < 0.05, FDR). Interestingly, 1283 of these genes were upregulated in strain sp. 008, while only 48 genes showed higher expression in strain KSM-B-3M (Figure 2A,B). This suggests a specific metabolic activity that defines KSM-B-3M dehydrogenation ability, possibly due to the loss or downregulation of certain pathways in this strain.

**Figure 3:**
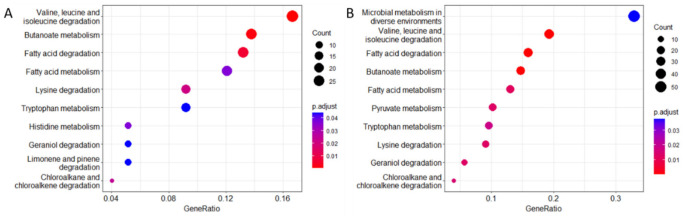
Dot-plot representing the ten most upregulated metabolic pathways in strain sp. 008 relative to KSM-B-3M. Strains were grown on *n*-hexadecane (**A**) and palmitic acid (**B**) for 7 days. The size of each dot corresponds to the number of genes related to this pathway, with dot color representing the statistical significance. One of the most significantly excessive pathways in strain sp. 008 is the fatty acid degradation pathway, along with the valine, leucine, and isoleucine degradation pathway and the butanoate metabolism pathway.

Notably, among the genes exhibiting significant and substantial excess expression in strain sp. 008 are those related to fatty acid degradation, including fatty acid CoA-ligase (WP_003942422.1), pyridine nucleotide-disulfide oxidoreductase (WP_042926002.1, WP_003942429.1), and aldehyde dehydrogenase (WP_003942430.1; Supplementary Table S2). Proteomic analysis corroborated these findings at the protein level; enrichment analysis identified four metabolic pathways significantly more active in strain sp. 008 compared to strain KSM-B-3M. The fatty acid degradation pathway was the most notably enriched among these pathways (adjusted *p*-value = 2.6 × 10^−4^), with ten genes showing higher expression in strain sp. 008 compared to strain KSM-B-3M (Supplementary Table S3, Supplementary Figure S2). With the latter strain demonstrating a unique biological activity of alkane dehydrogenation, we then investigated whether this yet unexplored pathway could be linked to a reduced fatty acid degradation activity in strain KSM-B-3M. The gene expression profile was therefore examined for both strains grown on either *n*-hexadecane or palmitic acid, which serves as an intermediate metabolic compound within the fatty acid degradation pathway. Our hypothesis was that if one of the enzymes responsible for the initial enzymatic steps in the pathway (converting alkane to fatty acid) is impaired in strain KSM-B-3M, the pathway might still function when initiated with palmitic acid as substrate. However, a comparative transcriptomic analysis of the two strains revealed once again that the fatty acid degradation pathway was among the most significantly upregulated pathways in strain sp. 008 for both *n*-hexadecane and palmitic acid (Figure 3) compared to strain KSM-B-3M. This appeared alongside upregulation of other pathways such as valine, leucine, and isoleucine degradation, and butanoate metabolism pathways, indicating additional variation in the metabolic activity between the strains. In addition, substantial strain-dependent variation in gene expression profiles was observed, with 90% of the variation in gene expression linked to differences between strains (PC1; Supplementary Figure S3). These observations suggest an inherent downregulation of the fatty acid degradation pathway in strain KSM-B-3M.

**Figure 4:**
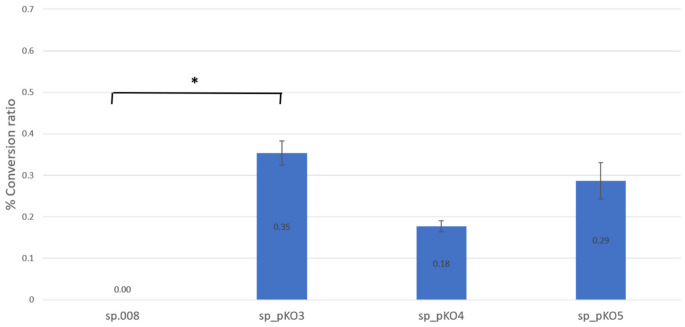
*n*-hexadecane conversion ratios obtained for strain sp. 008 and its cured clones sp_pKO3-5. Conversion ratios were measured after seven days of incubation in medium A supplemented with *n*-hexadecane. Values are averages of biological triplicates. *, *p* < 0.05.

Concurrent efforts to explore the downregulated pathway in strain KSM-B-3M involved genomic analysis. While sequence alignment of KSM-B-3M and sp. 008 chromosomes revealed a 99% identity, a significant genetic distinction was identified in their respective plasmids. Unlike strain sp. 008, which harbors three mega-plasmids, strain KSM-B-3M was found to possess two mega-plasmids that differ greatly from those of strain sp. 008. Notably, one of the absent plasmids in KSM-B-3M is pR8L1 (CP015203.1), which contains genes related to fatty acid degradation, including some of the most highly expressed genes in strain sp. 008 (Supplementary Table S2). Despite finding that all genes were redundant in the chromosomes of both strains, the marked deficiency of highly transcribed fatty acid degradation genes in strain KSM-B-3M could directly lead to an overall downregulation of the pathway in this strain (Supplementary Table S2). To directly investigate whether the absence of the pR8L1 plasmid and the subsequent downregulation of the fatty acid degradation pathway are linked to alkane dehydrogenation ability, strain sp. 008 was subjected to a plasmid-curing procedure using acridine orange treatment. This procedure resulted in the generation of several cured clones (clones sp_pKO3 to 5) that exhibited the loss of approximately 20% from the pR8L1 plasmid as determined by a set of PCR reactions roughly covering the plasmid. The DNA segment deleted from pR8L1 contained tens of genes, including acyl CoA ligase (WP_003942422.1, WP_081558941.1), aldehyde dehydrogenase (WP_003942430.1), and pyridine nucleotide disulfide oxidoreductase (WP_042926002.1), all of which are involved in the fatty acid degradation pathway. The ability of the cured clones to dehydrogenate alkanes was further evaluated. All clones demonstrated the capacity to dehydrogenate *n*-hexadecane to detectable levels, with conversion ratios ranging from 0.18% to 0.35% after 7 days, in contrast to the parental strain sp. 008 containing the intact pR8L1 plasmid that showed no detectable dehydrogenation ability (Figure 4). This suggests that partial plasmid loss enabled the cured clones to dehydrogenate *n*-hexadecane. At the next stage, we confirmed that the phenotypic difference between the cured clones and the wild-type strain sp. 008 originates from the partial plasmid deletion, rather than from other genetic variations in the chromosome or in the other plasmids, which may have occurred during the acridine orange treatment. Oxford Nanopore sequencing was therefore performed on the cured clone sp_pKO3, using the wild-type strain sp. 008 as a reference. Sequencing confirmed the deletion in plasmid pR8L1 and defined its boundaries at positions 1 and 127,890 with 133 deleted genes (Supplementary Table S4). No additional mutations were found in the genome of clone sp_pKO3. This supports the conclusion that the conversion phenotype of the cured clones is attributable to the loss of genes in plasmid pR8L1, and that metabolic pathways encoded on the pR8L1 plasmid compete with the alkane dehydrogenation ability. Variation in conversion ratios among the cured clones may suggest differences in fragment sizes and gene contents removed from each clone. The conversion ratio observed for strain KSM-B-3M after 7 days was 49.36%, still much higher compared to that of the cured clones, prompting further investigation into additional mechanisms related to *n*-hexadecane dehydrogenation.

**Figure 5:**
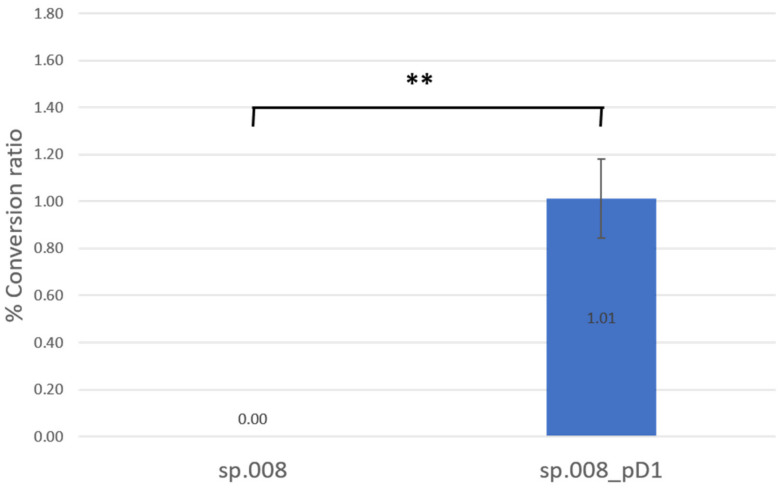
*n*-Hexadecane conversion ratios obtained for strain sp. 008, and the acyl-CoA desaturase overexpressing strain sp. 008_pD1. Conversion ratios were measured after seven days of incubation in medium A supplemented with *n*-hexadecane. Values are averages of 4–6 biological replicates. **, *p* < 0.01.

In addition to the impact of bacterial metabolic pathways, such as fatty acid degradation, as competitive with alkane dehydrogenation reaction, we delved into exploring which enzymes might be directly associated with alkane conversion. Both transcriptomic analyses revealed that the most abundant gene in strain KSM-B-3M was acyl-CoA desaturase (WP_003945297.1; Figure 2A, Supplementary Figure S4), showing a two-order-of-magnitude higher abundance compared to strain sp. 008. This was further validated by qPCR (RQ = 1438 for strain KSM-B-3M relative to strain sp. 008). Notably, the elevated expression of acyl-CoA desaturase correlated with high expression levels of two genes adjacent to it in the KSM-B-3M genome: ferredoxin reductase (WP_050656813.1) and a gene encoding a hypothetical protein (WP_003945295.1). Consequently, we defined the region containing these three genes as a putative operon (Figure 2A, Supplementary Figure S4). Indeed, acyl-CoA desaturase is recognized for catalyzing the conversion of saturated fatty acids to monounsaturated forms, with ferredoxin reductase serving as an electron donor essential for its function [39]. However, while palmitic acid is the typical substrate for acyl-CoA desaturase, alkanes had not been previously documented as such. In order to directly correlate acyl-CoA desaturase activity with alkane dehydrogenation, we proceeded towards overexpression of the acyl-CoA desaturase gene in strain sp. 008. As this strain naturally lacks detectable conversion ability, we examined whether the overexpression would result in a measurable ratio of alkane dehydrogenation. The overexpression was successful, as the transformed strain sp. 008_pD1 demonstrated acyl CoA-desaturase expression levels comparable to those of strain KSM-B-3M, as detected by qPCR (RQ = 1884 and 1438 for strains sp. 008_pD1 and KSM-B-3M, respectively, relative to strain sp. 008). Indeed, this overexpression resulted in a quantitative metabolic output, showing the formation of *cis*-hexadecene that reached a conversion ratio of 1.01% after 7 days, whereas the parental strain sp. 008 exhibited no detectable conversion (Figure 5). Efforts to overexpress the entire operon (namely the acyl-CoA desaturase, ferredoxin reductase, and the hypothetical protein) under the same constitutive promoter, in an attempt to further enhance the conversion ratio, yielded minimal levels of overexpression, suggesting a more intricate regulatory mechanism. Overall, the results clearly demonstrate a significant role of acyl-CoA desaturase in alkane dehydrogenation. It is noteworthy that the conversion ratio obtained for strain sp. 008 overexpressing acyl CoA-desaturase is still lower than that of strain KSM-B-3M, reaching 49.36%.

**Figure 6:**
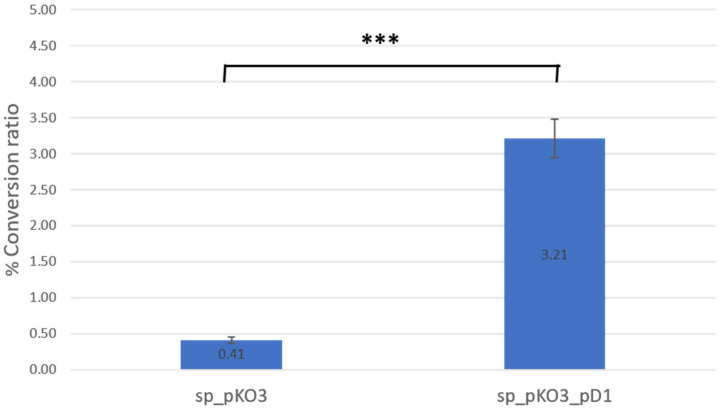
*n*-Hexadecane conversion ratios obtained for the cured clone before and after acyl-CoA desaturase overexpression. Sp_pKO3, the cured clone before acyl-CoA desaturase overexpression; sp_pKO3_pD1, the cured clone overexpressing acyl-CoA desaturase. Conversion ratios were measured after seven days of incubation in medium A supplemented with *n*-hexadecane. Values are averages of 6–12 biological replicates; ***, *p* < 0.001.

As both pR8L1 plasmid deletion and acyl-CoA desaturase overexpression contribute to the same phenotype of alkane dehydrogenation, we proceeded to investigate whether they directly influence each other. We therefore tested the expression levels of the acyl-CoA desaturase gene in pR8L1-cured clones, compared to the parental strain sp. 008 grown on *n*-hexadecane. No significant difference in acyl-CoA desaturase expression levels was observed among the strains, indicating that the deficiency of fatty acid degradation genes in the cured clones did not induce overexpression of acyl-CoA desaturase. This suggests that the downregulation of the fatty acid degradation pathway and the high expression levels of the acyl-CoA desaturase operon are independently regulated mechanisms, each separately contributing to alkane dehydrogenation ability. We therefore evaluated the combined impact of both mechanisms on alkane hydrogenation. A pR8L1-cured clone of strain sp. 008 (sp_pKO3, the cured clone showing the highest conversion ratio) was subjected to overexpression of the acyl-CoA desaturase gene. The resulting strain, sp_pKO3_pD1, exhibited clear overexpression of acyl CoA-desaturase as confirmed by qPCR, at a level comparable to that of strain KSM-B-3M (RQ = 2428 and 2761 for sp_pKO3_pD1 and strain KSM-B-3M, respectively, relative to the cured clone sp_pKO3). Indeed, a conversion ratio of 3.2% was achieved for the cured clone overexpressing acyl-CoA desaturase (sp_pKO3_PD1), which was significantly higher than that of the cured clone without acyl-CoA desaturase overexpression (Figure 6, *p* < 0.001). This indicates that both overexpression of acyl-CoA desaturase and the lack of pR8L1 plasmid containing fatty acid degradation genes provide two mechanisms that increase the ratio of alkane dehydrogenation in an additive manner.

**Figure 7:**
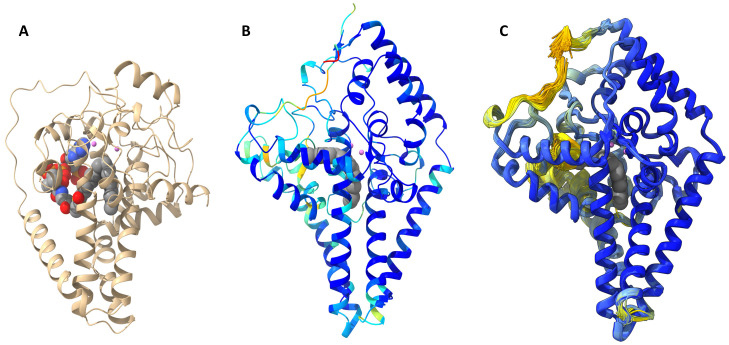
Comparison of Mouse CoA crystal structure (PDB 4ymk) and *Rhodococcus* acyl-CoA desaturase Rosetta All-atom model. (**A**), crystal structure of the distant homologue of mouse Stearoyl-Coenzyme A Desaturase 1 with its substrate Stearoyl Coenzyme A. Mouse protein in tan ribbon, ligands in spheres (C in grey, O in red and N in blue); (**B**), RoseTTA model of *Rhodococcus* acyl-CoA desaturase with the hexadecane and two Zn ions located in their putative binding site without any information provided about where it should be found. Colors (red, orange, yellow, cyan, blue) are given according to the AF color code, ligand in grey; (**C**), AlphaFold2 160 models of *Rhodococcus* acyl- CoA desaturase created by deep sampling methodology (see details in Section 2). Colors (red, orange, yellow, cyan, blue) are given according to the AF color code, from 0 to 100.

**Figure 8:**
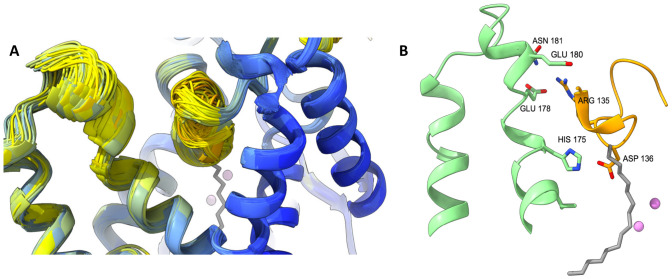
Catalytic site of *Rhodococcus* desaturases. (**A**), AlphaFold of 160 models showing the high flexibility of loops 130–140 and 170–200 (colored gold) encompassing the binding site; (**B**), 130–140 and 170–200 loops from the RoseTTA model, colored in orange and green, respectively, and showing electrostatic interactions between residues on both loops. Colors (red, orange, yellow, cyan, blue) are given according to the AF color code, from 0 to 100.

After confirming the involvement of these two mechanisms in alkane dehydrogenation, we further investigated the role of acyl-CoA desaturase in the dehydrogenation reaction. Specifically, we modeled whether the enzyme could directly bind to *n*-hexadecane and convert it into *cis*-hexadecene. The 3D structure of the *Rhodococcus* acyl-CoA desaturase was therefore predicted, using the RoseTTAFold All-Atom tool [29]. The RoseTTA model was obtained with the hexadecane as the proposed substrate and two Zn ions located in their putative binding site. To validate the model, we extensively studied and compared it with the closest available homologue, the Mouse Stearoyl-Coenzyme A Desaturase 1 (Mouse CoA for short (PDB 4ymk) [40]. Comparison of the Mouse CoA crystal structure containing its substrate Stearoyl Coenzyme A, with the high-quality RoseTTA model of *Rhodococcus* acyl CoA desaturase and 160 models made by AlphaFold2 (see details in Section 2) demonstrates that the *Rhodococcus* models have a similar organization when compared to Mouse Stearoyl-Coenzyme A Desaturase 1 (Figure 7). This similarity was more noticeable on the transmembrane (TM) region (Supplementary Figure S5A), with a global RMSD of 4.48, indicating a similar fold despite the low sequence identity of these two homologues (17%). The binding site is significantly more structurally conserved, with an RMSD of 0.45 for the residues within 10 Å of the ligands. The multiple AlphaFold models obtained by sub-sampling the MSA (see Methods) further show several flexible regions (colored gold to orange), with lower pLDDT values. These areas correspond to the N- and C- terminus, usually more flexible, and two loops close to the putative hexadecane binding site (Figure 7C and Figure 8A). When zooming into the binding site (Figure 8), the two flexible loops (130–140 and 170–200) appear to encompass the binding site, being very close to the ligand. We speculate that they serve as a gate to the entrance and exit of the ligand to the catalytic pocket, as in both the RoseTTA and AlphaFold models, the way to the binding pocket is completely closed to the substrate, requiring a change of conformation to allow the substrate entrance. Furthermore, there are a couple of strong polar interactions between Arg135 of the first loop and Asn181, Glu180, and Glu178 of the second loop, and between Asp136 of the first loop and His175 of the second loop, which could be the key to maneuver the opening of the entrance to the binding site.

**Figure 9:**
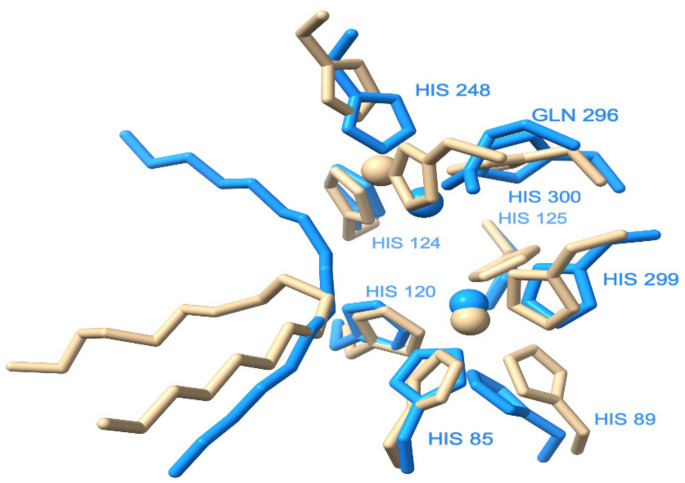
Structural alignment of catalytic site residues in the Mouse structure and *Rhodococcus* acyl-CoA desaturase model. Hexadecane, ions, and histidine residues of the *Rhodococcus* model, which are involved in Zn binding, are shown in blue, while the same corresponding residues, substrate, and ions in the crystal structure of Mouse CoA are colored tan.

Comparison of the coordination sphere of the Zn ions on the Mouse Desaturase structure with the same region of the *Rhodococcus* model (Figure 9) revealed that structural similarity is very high, with a local Cα RMSD of 1.6 Å, indicating a very similar organization of the catalytic center. The latter includes eight fully conserved His residues (both structurally and sequentially), an additional His in the Mouse desaturase replaced by a Gln in the *Rhodococcus* desaturase, and a similar orientation of the carbon atoms of both ligands with respect to the two Zn ions. This highly conserved Figure 1:

Comparison of the coordination sphere of the Zn ions on the Mouse Desaturase structure with the same region of the *Rhodococcus* model (Figure 9) revealed that structural similarity is very high, with a local Cα RMSD of 1.6 Å, indicating a very similar organization of the catalytic center. The latter includes eight fully conserved His residues (both structurally and sequentially), an additional His in the Mouse desaturase replaced by a Gln in the *Rhodococcus* desaturase, and a similar orientation of the carbon atoms of both ligands with respect to the two Zn ions. This highly conserved organization around the bimetal center, despite a different global structural organization, supports a conserved binding site with a similar putative catalytic role of the conserved histidine residues, and the correctness of the approximate position of the hexadecane with the catalytic site area. Importantly, the carbon atoms positioned in the acyl chain kink within the tunnel fit to the position of double bond insertion by strain KSM-3BM, yielding *cis*-7- and *cis*-8-hexadecene. Furthermore, the hydrophobic profile of the TM binding area (Supplementary Figure S5A) and the pocket around the hexadecane (Supplementary Figure S5B) show a similar hydropathicity pattern of both the Mouse and *Rhodococcus* molecules, also indicating a similar localization and similar functional pattern.

This analysis suggests that acyl-CoA desaturase, which is highly overexpressed in strain KSM-3BM, may directly convert *n*-hexadecane to *cis*-hexadecene. This is proposed in addition to its classic mechanism of action, acting on acyl-CoA thioesters. Typically, the expression of this enzyme is repressed when its usual substrate, fatty acids, is present, as monounsaturated fatty acids act as negative signaling molecules [41]. However, we observed higher acyl-CoA desaturase expression levels under alkanes compared to fatty acid (Supplementary Figures S6 and S7; see Supplementary Materials for detailed information). This observation suggests that, unlike monounsaturated fatty acids, alkenes may not function as negative signaling molecules for acyl-CoA desaturase.

In order to directly assess whether acyl-CoA desaturase catalyzes the conversion of *n*-hexadecane to *cis*-hexadecene, we established an in vitro enzymatic assay using recombinant murine and *Rhodococcal* acyl-CoA desaturase enzymes. Under the conditions tested, no conversion of *n*-hexadecane to *cis*-hexadecene was detected. This lack of activity may reflect suboptimal reaction conditions or the requirement for additional yet unidentified protein components. Further investigation is needed to clarify these possibilities and to establish conditions that support this in vitro reaction.

All in all, we identified two biological mechanisms that cumulatively contribute to the alkane dehydrogenation capability of strain KSM-3BM: downregulation of the fatty acid degradation pathway, which competes with alkane dehydrogenation; and overexpression of acyl-CoA desaturase, which is proposed to directly dehydrogenate alkanes, converting them to alkenes.

## 4. Discussion

Site-selective alkane functionalization and the production of corresponding alkenes hold significant industrial importance. Following our previous work, leveraging *Rhodococcus* sp. KSM-B-3M efficient alkane dehydrogenation capabilities to generate an array of alkyl derivatives [8], we have delved into the biological mechanisms standing behind this unique alkane dehydrogenation ability. Transcriptional analysis revealed a unique gene expression profile in strain KSM-B-3M when grown on *n*-hexadecane, in contrast to the closely related reference strain sp. 008, which does not exhibit any detectable ability to convert alkanes (Figure 1 and Figure 2, Supplementary Figures S3 and S4). Notably, strain sp. 008 exhibited excessive expression of genes associated with the fatty acid degradation pathway (Figure 3).

Fatty acid degradation is a well-established pathway utilized for the oxidation of fatty acids [42,43], being a part of the rhodococci toolkit for energy harvest [9]. This pathway is further extended for alkane degradation, leveraging rhodococci’s capability to form stable biofilms on hydrophobic surfaces [44,45]. A similar enrichment of the fatty acid degradation pathway in strain sp. 008, compared to strain KSM-B-3M, was observed when both strains were cultured on palmitic acid, an intermediate compound of the pathway from which the β-oxidation cycle initiates [42]. This prompted an investigation into the intrinsic mechanisms underlying the downregulated fatty acid degradation in strain KSM-B-3M, potentially linked to its unique alkane dehydrogenation capability. Surprisingly, genomic analysis unveiled significant disparities in the mega-plasmids carried by the two strains; strain KSM-B-3M harbors two plasmids, which are distinct from the three plasmids carried by strain sp. 008. Notably, several genes encoding the fatty acid degradation pathway were identified on one of strain sp. 008’s plasmids (pR8L1), which is absent in strain KSM-B-3M. This finding aligns with existing literature describing specific *Rhodococcus* plasmids, such as pREC1 in *Rhodococcus erythropolis* strain PR4, that carry genes associated with the fatty acid degradation pathway [46]. Among the prominently expressed fatty acid degradation genes in strain sp. 008 is the fatty acid CoA-ligase (WP_003942422.1), whose orthologs, FAA1 and FAT1, have previously been shown to be upregulated by n-alkanes [47]. Despite the absence of the pR8L1 plasmid containing multiple fatty acid degradation genes from the KSM-B-3M genome (Supplementary Table S2), their functional annotations suggest that these genes are redundant in both genomes. Relying solely on the functional annotations may not conclusively determine true gene redundancy [48], as is particularly evident for acyl-CoA ligase orthologs [47]. Regardless, deletions of highly expressed fatty acid degradation genes in strain KSM-B-3M are expected to result in downregulation of the entire fatty acid degradation pathway. This downregulation was further confirmed through protein analysis (Supplementary Figure S2, Supplementary Table S3).

To directly investigate the potential link between the absence of the pR8L1 plasmid and alkane dehydrogenation capability, we conducted a plasmid curing process on the control strain sp. 008. This procedure led to the deletion of a large DNA fragment, of approximately 140,000 bp (20% of the plasmid). This fragment included, among others, four genes involved in fatty acid degradation, notably the highly expressed acyl CoA ligase (WP_003942422.1) and pyridine nucleotide-disulfide oxidoreductase (WP_042926002.1). Remarkably, the cured clones were able to produce *cis*-hexadecene at detectable levels (Figure 4), indicating that the pR8L1 plasmid, housing genes responsible for the fatty acid degradation pathway, acts as a competitor to alkane dehydrogenation. The absence of this plasmid in strain KSM-B-3M appears to facilitate alternative metabolic activities, such as alkane dehydrogenation. The capability of strain KSM-B-3M to operate without certain fatty acid degradation genes aligns with findings in *Rhodococcus equi*, where genes associated with lipid metabolism are enriched in the *R. equi* accessory genome compared to its core genome [49], and may constitute a non-essential gene set.

Beyond demonstrating a clear role for the absence of fatty acid degradation-encoding plasmids in alkane dehydrogenation, our investigation focused on identifying the enzymatic activities directly associated with alkane dehydrogenation. Repeatedly, the most highly expressed gene in the transcriptome of strain KSM-B-3M was acyl-CoA desaturase (WP_003945297.1; Figure 2, Supplementary Figure S4). Acyl CoA desaturase, also known as stearoyl-CoA desaturase (SCD), is recognized for its ability to catalyze the dehydrogenation of saturated acyl-CoA chains, favoring stearoyl-CoA or palmitoyl-CoA [40,50], to generate monounsaturated fatty acids. This reaction is coupled with an electron transfer chain; in mammals, it commences with cytochrome b5 reductase, proceeds to cytochrome b5, and culminates in SCD1 [51]. In plants and *Bacillus subtilis*, ferredoxin, ferredoxin reductase, and flavodoxins have been identified as components of this electron transfer chain [39,51]. Indeed, our results reveal that in addition to the high expression of acyl-CoA desaturase, both ferredoxin reductase (WP_050656813.1) and a gene encoding a hypothetical protein (WP_003945295.1) were significantly expressed (Figure 2, Figure S4). Since these genes are located adjacent to acyl-CoA desaturase in the KSM-B-3M genome, we have classified all three genes as a putative operon.

Previous examination of desaturase structure, focused on mouse and human SCD, unveiled the presence of a V-shaped tunnel within the protein. This tunnel accommodates the fatty acyl chain, with its inflection point situated near the protein’s active site, housing two ferrous ions. The dimensions of the tunnel are such that carbons 9 and 10 of the acyl chain align with the inflection point, positioning their two hydrogen atoms in a *cis* configuration close to the ferrous ions. This configuration ensures regiospecific and stereospecific double bond formation [40,50]. Notably, the primary isomer produced by strain KSM-B-3M featured a double bond at position C9, resulting in *cis*-7-hexadecene [8], indicating direct involvement of acyl-CoA desaturase in double bond formation. The direct act of the enzyme on *n*-hexadecane has been confirmed as possible through 3D structure modeling (Figure 7, Figure 8 and Figure 9). The model indeed located the acyl chain inside a hydrophobic tunnel with positions of carbon atoms fitting to the position of double bond formation by strain KSM-3BM in the tunnel kink. The catalytic pocket, composed of a bimetal center surrounded by histidine residues, demonstrated high homology compared to the closest available Mouse Stearoyl-Coenzyme A Desaturase. Two flexible loops appeared to close the substrate binding pocket, and a couple of strong polar interactions between amino acid residues may serve as the key to maneuver the opening of the entrance to the binding site. This support of a key role for acyl-CoA desaturase in alkane dehydrogenation has been ultimately reinforced by the overexpression of acyl-CoA desaturase in the control strain sp. 008, which led to the formation of *cis*-hexadecene (Figure 5).

Previous studies have associated acyl-CoA desaturase genes with increased alkene production in flies [52,53,54,55]; however, these described desaturase genes have been reported to utilize stearoyl-CoA precursors for alkene production through decarboxylation [55,56]. Our results necessitate the use of an alternative alkene formation mechanism, such as direct dehydrogenation by acyl-CoA desaturase, as the substrate and the product share the same carbon chain length. Our work, therefore, represents the first instance of *cis*-hexadecene formation from *n*-hexadecane through a biological system.

The gathered results have confirmed a direct involvement of both acyl-CoA desaturase overexpression and the deletion of pR8L1 plasmid genes in the conversion of *n*-hexadecane to *cis*-hexadecene. As both contribute to the same phenotype of alkane dehydrogenation, we proceeded to investigate whether they directly influence each other, and whether the deletion of fatty acid degradation genes could directly result in overexpression of the acyl-CoA desaturase. Notably, pR8L1-cured clones did not exhibit increased acyl-CoA desaturase expression, suggesting that the two factors contribute to alkane dehydrogenation in a cumulative manner. Indeed, the overexpression of acyl-CoA desaturase in the pR8L1-cured clone significantly enhanced conversion ability (Figure 6); this was additive to the increase in conversion ratio of the cured clone compared to strain sp. 008 (Figure 5). This validates the distinct impact of each feature on the alkane conversion phenotype, being a quantitative trait. The fact that strain KSM-B-3M still exhibits notably higher conversion efficiency could be attributed to various reasons, such as incomplete curing of the pR8L1 plasmid or other unexplored mechanisms, calling for further extension of our understanding in the frame of future studies.

## 5. Conclusions

Our research sheds light on the mechanism behind the alkane dehydrogenation ability exhibited by *Rhodococcus* sp. KSM-B-3M, a process of significant industrial importance. Acyl-CoA desaturase was recognized as a pivotal component in alkane dehydrogenation, along with the downregulation of the fatty acid degradation pathway, both contributing to the reaction in an additive manner. Together with future studies required to fully elucidate this unique biotransformation, this study has paved the way for advancing industrial production of alkyl derivatives, being crucial intermediates in the pharmaceutical industry, through efficient and economically viable biological processes.

## Data Availability

The genome sequence of *Rhodococcus* sp. KSM-B-3M, as well as the RNA-seq data generated in this study, have been deposited in the NCBI under BioProject PRJNA1281853.

## Supplementary Materials

The following supporting information can be downloaded at: https://www.mdpi.com/article/10.3390/microorganisms14061252/s1, Table S1: List of primers used in the study; Figure S1: Phylogenetic tree of selected Rhodococcus strains; Table S2: Gene distribution and number of reads for fatty acid degradation genes along the KSM-B-3M and sp. 008 genomes; Table S3: Go Terms Enrichment of proteins expressed significantly higher in strain sp. 008 compared to strain KSM-B-3M, as identified by proteomic analysis; Figure S2: Down-regulation of the fatty acid degradation pathway in strain KSM-B-3M as observed by the DAVID database based on a KEGG pathway map; Table S4: List of genes cured from plasmid pR8L1 (CP015203.1) of clones sp_pKO3-5 (derivates of strain sp.008); Figure S3: Principal component analysis (PCA) demonstrating the transcriptomic profiles of strains KSM-B-3M and sp. 008 grown on n-hexadecane or palmitic acid; Figure S4: Differential gene expression between strains KSM-B-3M and sp. 008; Table S5: Substrates divided according to their dehydrogenation productivity by strain KSM-B-3M; Figure S5: The hydrophobicity pattern of Mouse and *Rhodococcus* desaturases; Figure S6: Expression levels of acyl-CoA desaturase, ferredoxin reductase and the gene encodes for hypothetical protein as obtained on n-hexadecane and palmitic acid; Figure S7: Acyl-CoA desaturase expression in strain KSM-B-3M grown on alkanes (blue bars) vs. saturated fatty acids (green bars) as determined by qPCR analysis.
