# Supplementary material for: Insights into the Mechanism Underlying the Alkane Dehydrogenation Capability of Rhodococcus sp. KSM-B-3M
Source: Microorganisms. 2026 Jun 2;14(6):1252. doi: 10.3390/microorganisms14061252 (PMC13304129; doi:10.3390/microorganisms14061252)
Supplement: Supplementary file 1 [file microorganisms-14-01252-s001.zip › microorganisms-4314607-supplementary.pdf]

Supplementary materials

Table S1: List of primers used in the study

Primer name	Target / Position	Nucleotide sequence (5' to 3')	Amplicon length (bp)	Annealing temperature
16S F rRNA gene	16S gene	F: TCGTCGGTGATTGTTCATTC	575	60
16S R rRNA gene		R: TCCCTGCTGAAAGAGGTTTA		
Desaturase_F	CP0112749.1:6433937-6433959	F: CAAACCGTACTTCCCACACAATT	107	60
Desaturase_R	CP0112749.1:6434024-6434043	R: GCAACCTCTCGCACCAGATC		
Ferredoxin_F	CP0112749.1:6435127-6435147	F: GGTGCAGGAGAAGCAGATTCC	132	60
Ferredoxin_R	CP0112749.1:6435242-6435258	R: GGCGGCGAGGTGTCATT		
Hypo_F	CP0112749.1:6433566-6433584	F: TGTCGTCGTGCTCGATGGT	80	60
Hypo_R	CP0112749.1:64333627-643345	R: CGGAATCGAGATGCGTGAA		
Ligase_RT F	CP015203.1:18346-183365	F: GGGTGGAGCTGAGGATTGTC	152	64
Ligase_RT F	CP015203.1:18478-18497	R: GCGTAACCACCCTTCCTGAA		
Ligase1_F	CP015203.1:17861-17880	F: GACGTACTTGCATTCGATGG	685	62
Ligase1_R	CP015203.1:18527-18545	R: GGTCAGCGTCACGAAATTG		
pR8C1_1 F	CP015204.1:12419-12438	F: AGCGGCTTTGTTTCCTACGA	950	63
pR8C1_1 R	CP015204.1:13349-13368	R: GAGAACTGTCGTTGGTCGGT		
pR8C1_2 F	CP015204.1:79629-79648	F: AGAAGACGATCTGGAGCCCT	867	63

pR8C1_2 R	CP015204.1:80476-80495	R: AACGACTCAACGACACCCTC		
pR8C1_3 F	CP015204.1:42064-42083	F: CATGCTCAATCGTTGCGGAG	662	63
pR8C1_3 R	CP015204.1:42706-42725	R: GACCTGGTAGATCTTCGGCG		
pR8C2_1 F	CP015205.1:3576-3595	F: AGTGGGCAGCGATTCTTCAA	870	63
pR8C2_1 R	CP015205.1:4426-4445	R: CCTCCGTTGTAACCGTGTCA		
pR8C2_2 F	CP015205.1:23320-23339	F: GCAGCGATCCCTTACTCCTC	897	63
pR8C2_2 R	CP015205.1:24197-24216	R: ACCTGCACAGTCTTGACTCG		
pR8C2_3 F	CP015205.1:47069-47088	F: CTACGGTCTCGATGCTCCAC	843	62
pR8C2_3 R	CP015205.1:47892-47911	R: GAGAAACAGACCTCCGCACA		
PR8L1-1 F	CP015203.1:232069-232088	F: GCGATGGAACGATCAACTGC	540	62
PR8L1-1 R	CP015203.1:232589-232608	R: AGCCCCCAGTAGTAGGTGAG		
PR8L1-2 F	CP015203.1:447137-447156	F: CGAAACGAAGCACAGCAACA	429	62
PR8L1-2 R	CP015203.1:447546-447565	R: CGCATCGAACTACCCTCTCC		
PR8L1-3 F	CP015203.1:3473-3492	F: CCTGCGCAATATCGGACTCT	652	64
PR8L1-3 R	CP015203.1:4105-4124	R: TCCCTGACGTGTCCCTCTAG		
PR8L1-4 F	CP015203.1:9738-9757	F: TTGTGTTGCTGATCGTCGGA	657	64

PR8L1-4 R	CP015203.1:10375-103393	R: ACAAAGGAGGGCGTTGGTA		
PR8L1-5 F	CP015203.1:89709-89728	F: GAAACTTGTCCGCACCCAAC	497	64
PR8L1-5 R	CP015203.1:90186-90205	R: CGAAAGCAATGTACGCCTCG		
PR8L1-6 F	CP015203.1:140173-140192	F: CAGTGGGTGTGAAGCGTTTG	522	64
PR8L1-6 R	CP015203.1:140675-140694	R: AACAGACCACCATCGACACC		
PR8L1-7 F	CP015203.1:689744-689763	F: AGACCCATGCCCTTAACGTG	766	64
PR8L1-7 R	CP015203.1:690490-690509	R: CGCGAGGATTCCCGTAATGA		
Desaturase_NdeI_F	Acyl CoA desaturase over expression	F: CCTATTCATATGTTTGGACTTTCACTTCC	1173	62
Desaturase_ApaI_R		R: TGCTTAGGGCCCTCAGGCTGCAGTTCGCTCGC		
Operon_NdeI_F	Whole putative operon over expression	CCTATTCATATGGCTGCTCCACATCAGAT	2517	62
Operon_ApaI_R		TGCTTAGGGCCCTCAACGCCAGTCTTCAGGTG		
pDD120_seq-F	Verification of acyl CoA desaturase transformants	F: CGGGCTCTAACACGTCCTAG	pDD120 2158bp pD1 1338bp	63
pDD120_seq-R		R: TCGTACGCTAGTTGCAGATCA		

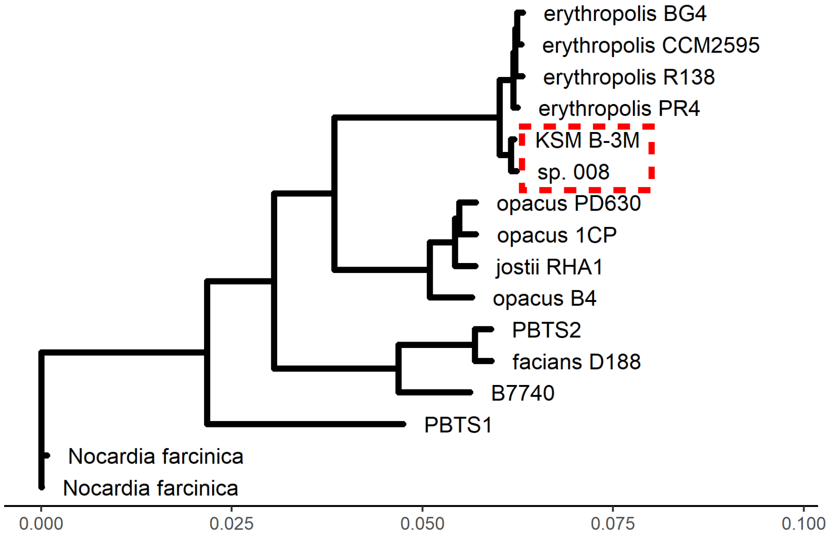

Figure S1: Phylogenetic tree of selected *Rhodococcus* strains. The tree was constructed based on genome sequences, indicating that *Rhodococcus* sp. 008 is the genetically closest to strain KSM-B-3M compared to the tested strains.

Table S2: Gene distribution and number of reads for fatty acid degradation genes along the KSM-B-3M and sp. 008 genomes. Gene positions are given according to the sp. 008 genome. Number of RNAseq reads are given as an average of biological triplicates. The comprehensive gene list was constructed by genes extracted from the KEGG database with additional entries added manually according to functional annotations. Excessive genes in strain sp. 008 transcriptome are marked in bold. CP012749.1 correspond to sp.008 chromosome. CP015203.1 and CP015204.1 correspond to pR8L1 and pR8C1, respectively.

Product	Name	Gene_position	Average read numbers (RNAseq)			
			Strain sp. 008		Strain KSM-B-3M	
			n-hexadecane	Palmitic acid	*n*-hexadecane	Palmitic acid
3-hydroxybutyryl-CoA_dehydrogenase	WP_030536170.1	CP012749.1:1809339-1810199	966.90	67.98	272.27	120.38
3-hydroxyacyl-CoA_dehydrogenase	WP_003943799.1	CP012749.1:2697729-2699870	202.76	254.71	99.70	310.25
acetyl-CoA acyltransferase	WP_065351439.1	CP012749.1:1275583-1276713	6.46	17.52	9.69	13.49

acetyl-CoA acyltransferase	WP_054825852.1	CP012749.1:2410267-2411457	143.67	194.83	1172.90	59.01
acetyl-CoA acyltransferase	WP_042926492.1	CP015203.1:108174-109319	105.35	137.01	0.00	0.00
acetyl-CoA_acetyltransferase	WP_007732681.1	CP012749.1:1177551-1178714	0.86	0.71	3.74	6.43
acetyl-CoA C-acetyltransferase	WP_064114110.1	CP012749.1:1265556-1266752	17.81	11.37	27.76	17.96
acetyl-CoA C-acetyltransferase	WP_064114209.1	CP012749.1:1291588-1292808	20.10	54.18	13.09	13.77
acetyl-CoA C-acetyltransferase	WP_030536550.1	CP012749.1:1391789-1393000	4.51	7.87	7.72	19.18
acetyl-CoA C-acetyltransferase	WP_065352796.1	CP012749.1:1823800-1824957	11.49	8.61	10.86	6.07
acetyl-CoA_acetyltransferase	WP_065351708.1	CP012749.1:2267263-2268372	7.72	27.40	0.00	0.00
acetyl-CoA_acetyltransferase	WP_007731553.1	CP012749.1:2696469-2697683	201.76	270.46	84.09	216.11
acetyl-CoA_acetyltransferase	WP_054188603.1	CP012749.1:2715966-2717096	4.56	4.03	12.58	5.70
acetyl-CoA C-acetyltransferase	WP_007734192.1	CP012749.1:3173332-3174480	105.66	125.69	106.95	53.49
acetyl-CoA_acetyltransferase	WP_065351952.1	CP012749.1:3207017-3208180	6.62	13.56	11.91	22.27
acetyl-CoA C-acetyltransferase	WP_007734272.1	CP012749.1:3211674-3212855	2.77	0.00	1.63	11.68
acetyl-CoA C-acetyltransferase	WP_019744600.1	CP012749.1:337083-338300	101.57	111.61	139.49	309.49
acetyl-CoA C-acetyltransferase	WP_065351999.1	CP012749.1:3433520-3434875	150.35	447.18	152.56	636.37
acetyl-CoA C-acetyltransferase	WP_065352025.1	CP012749.1:3529919-3531118	10.84	62.57	30.26	158.12
acetyl-CoA C-acetyltransferase	WP_030534916.1	CP012749.1:586266-587498	4.05	51.90	22.37	45.83
acetyl-CoA C-acetyltransferase	WP_030534917.1	CP012749.1:587622-588833	0.88	2.64	2.58	6.16
acetyl-CoA C-acetyltransferase	WP_065352707.1	CP012749.1:6489066-6490259	18.23	16.88	7.94	19.17
acetyl-CoA acetyltransferase	WP_042920147.1	CP015203.1:105766-106962	348.37	73.58	0.31	1.83
acetyl-CoA C-acetyltransferase	WP_058227884.1	CP015203.1:336171-337397	52.84	54.42	0.00	0.00
acetyl-CoA C-acetyltransferase	WP_058228032.1	CP015203.1:611433-612584	2.94	10.92	0.00	0.00
acetyl-CoA_acetyltransferase	WP_058228044.1	CP015203.1:628164-629357	2.95	11.41	0.00	0.00
acetyl-CoA C-acetyltransferase	WP_011331211.1	CP015204.1:56716-57870	44.82	60.05	0.00	0.00
acetyl-CoA acyltransferase	WP_065351439.1	CP012749.1:1275583-1276713	6.46	17.52	9.69	13.49
acetyl-CoA_C-acyltransferase	WP_007730836.1	CP012749.1:1600297-1601496	8.76	18.79	6.08	48.00
acetyl-CoA_C-acyltransferase	WP_065351561.1	CP012749.1:1764415-1765608	15.53	24.60	19.57	21.19
acetyl-CoA acyltransferase	WP_054825852.1	CP012749.1:2410267-2411457	143.67	194.83	1172.90	59.01
acetyl-CoA_C-acyltransferase	WP_007731825.1	CP012749.1:2480374-2481555	93.88	146.78	99.52	248.16

acetyl-CoA_C-acyltransferase	WP_042452159.1	CP012749.1:3775675-3776844	8.29	9.13	13.09	19.64
acetyl-CoA_C-acyltransferase	WP_003942598.1	CP012749.1:5269741-5270961	2418.11	241.73	2825.42	2799.54
acetyl-CoA acyltransferase	WP_042926492.1	CP015203.1:108174-109319	105.35	137.01	0.00	0.00
acetyl-CoA_C-acyltransferase	WP_065353039.1	CP015204.1:50543-51718	321.61	288.44	0.00	0.19
acyl-CoA_dehydrogenase	WP_065351366.1	CP012749.1:1001644-1002708	13.15	9.84	47.61	25.03
acyl-CoA_dehydrogenase	WP_065351392.1	CP012749.1:1099457-1100668	2.88	7.19	9.09	1.03
acyl-CoA_dehydrogenase	WP_003940617.1	CP012749.1:1272632-1273789	10.49	408.46	20.98	93.65
acyl-CoA_dehydrogenase	WP_003940162.1	CP012749.1:1325592-1326803	33.43	28.14	32.09	39.92
acyl-CoA_dehydrogenase	WP_019745701.1	CP012749.1:1607528-1608748	13.66	122.16	37.59	203.17
acyl-CoA_dehydrogenase	WP_007731824.1	CP012749.1:2481558-2482709	54.15	54.61	94.79	145.90
acyl-CoA_dehydrogenase	WP_065351822.1	CP012749.1:2736391-2738172	57.47	88.15	130.42	54.68
acyl-CoA_dehydrogenase	WP_042448906.1	CP012749.1:391371-392561	34.74	54.91	128.53	13.34
acyl-CoA_dehydrogenase	WP_007728404.1	CP012749.1:4106153-4107301	13.26	34.78	15.32	32.49
acyl-CoA_dehydrogenase	WP_003942363.1	CP012749.1:4646906-4648111	215.47	103.47	335.29	279.14
acyl-CoA_dehydrogenase	WP_007735018.1	CP012749.1:5032550-5034280	184.08	67.86	212.75	275.86
acyl-CoA_dehydrogenase	WP_007735194.1	CP012749.1:5200416-5201558	56.14	150.90	71.10	120.58
acyl-CoA_dehydrogenase	WP_065352695.1	CP012749.1:6408460-6409659	6.52	9.47	8.89	1.82
acyl-CoA_dehydrogenase	WP_065352756.1	CP012749.1:753312-754490	8.91	15.62	27.63	20.25
acyl-CoA_dehydrogenase	WP_003939770.1	CP012749.1:955810-956982	17.90	19.49	51.58	22.56
acyl-CoA_dehydrogenase	WP_058227854.1	CP015203.1:295441-296595	207.06	171.68	0.00	0.00
acyl-CoA_dehydrogenase	WP_058227928.1	CP015203.1:337873-339009	54.35	75.92	0.00	0.00
acyl-CoA_dehydrogenase	WP_074447131.1	CP015203.1:353184-354344	15.90	83.76	0.00	0.00
acyl-CoA_dehydrogenase	WP_058228500.1	CP015203.1:379004-380152	18.62	17.25	0.00	0.00
acyl-CoA_dehydrogenase	WP_058228031.1	CP015203.1:610281-611429	50.00	164.87	0.00	0.00
acyl-CoA_dehydrogenase	WP_058228096.1	CP015203.1:630213-631382	4.53	15.99	0.00	0.00
acyl-CoA_dehydrogenase	WP_058228051.1	CP015203.1:640545-641813	60.64	44.58	0.00	0.00
acyl-CoA_dehydrogenase	WP_058228077.1	CP015203.1:680010-681122	9.41	11.59	0.00	0.00
acyl-CoA dehydrogenase	WP_058228078.1	CP015203.1:681137-682327	29.37	33.73	0.00	0.00
acyl-CoA_dehydrogenase	WP_011331214.1	CP015204.1:54187-55347	37.77	87.76	0.00	0.00
acyl-CoA_oxidase	WP_024487810.1	CP012749.1:4019001-4020932	309.17	139.12	442.18	701.84
acyl-CoA_synthetase	WP_065351530.1	CP012749.1:1616858-1618513	12.96	14.36	7.15	12.59
acyl-CoA_synthetase	WP_065351732.1	CP012749.1:2340692-2342341	28.55	61.06	18.70	24.31

acyl-CoA_synthetase	WP_065351747.1	CP012749.1:2387688-2389385	6.01	45.07	18.35	60.95
aldehyde_dehydrogenase	WP_065351609.1	CP012749.1:1897966-1899441	4.09	12.48	21.75	7.71
aldehyde_dehydrogenase_family_protein	WP_007732506.1	CP012749.1:2842641-2844065	191.86	425.82	179.78	192.44
aldehyde_dehydrogenase	WP_003945530.1	CP012749.1:3409371-3410816	0.35	3.17	3.66	14.31
aldehyde_dehydrogenase	WP_065352526.1	CP012749.1:5831974-5833440	2.60	16.48	1.74	14.48
**aldehyde_dehydrogenase**	**WP_003942430.1**	**CP015203.1:13704-15233**	**3141.68**	**58.11**	**0.14**	**0.40**
aldehyde dehydrogenase (NAD+)	WP_058227866.1	CP015203.1:311613-313088	27.73	85.36	0.00	0.00
aldehyde_dehydrogenase	WP_058227896.1	CP015203.1: 350149-351591	6.16	61.95	0.00	0.00
aldehyde_dehydrogenase	WP_058227906.1	CP015203.1:363304-364836	33.03	172.58	0.00	0.00
aldehyde_dehydrogenase	WP_058227908.1	CP015203.1:366514-367995	6.56	42.09	0.00	0.00
aldehyde_dehydrogenase	WP_058228579.1	CP015203.1:401917-403470	131.72	123.52	0.00	0.00
aldehyde_dehydrogenase	WP_058228534.1	CP015203.1:558992-560440	76.94	415.85	0.00	0.00
betaine-aldehyde_dehydrogenase	WP_042920093.1	CP015203.1:78646-80130	181.13	133.62	0.00	0.00
alkane_1-monooxygenase	WP_007730881.1	CP012749.1:1577110-1578276	77.15	21.37	151.33	77.74
alkane_1-monooxygenase	WP_003945652.1	CP012749.1:3104468-3105643	8345.35	40.86	10796.14	25.89
alkane_1-monooxygenase	WP_054826870.1	CP012749.1:3581544-3582695	1.10	11.25	7.50	26.50
alkane_1-monooxygenase	WP_065352226.1	CP012749.1:4559976-4561202	564.31	162.40	409.74	127.24
alkane_1-monooxygenase	WP_030535413.1	CP012749.1:4885067-4886227	36.14	136.93	134.78	254.44
FAD-dependent_monooxygenase	WP_063892155.1	CP015203.1:669681-670883	44.50	52.79	0.00	0.00
enoyl-CoA_hydratase	WP_003944701.1	CP012749.1:4839723-4840502	43.90	53.90	27.35	64.06
enoyl-CoA_hydratase	WP_003941374.1	CP012749.1:6556535-6557311	142.23	187.41	874.40	264.90
FAD-dependent_oxidoreductase	WP_065352689.1	CP012749.1:6380415-6381650	192.44	337.45	107.42	114.69
long-chain-fatty-acid--CoA_ligase	WP_042448537.1	CP012749.1:1277529-1279229	1.55	1.04	6.16	5.74
long-chain_fatty_acid--CoA_ligase	WP_042450345.1	CP012749.1:160834-162621	458.29	614.63	307.25	559.69
fatty-acid--CoA_ligase	WP_007730820.1	CP012749.1:1611882-1613384	6.54	9.65	10.41	23.04
long-chain_fatty_acid--CoA_ligase	WP_065351812.1	CP012749.1:2680339-2681928	5.13	6.64	25.71	5.27
long-chain_fatty_acid--CoA_ligase	WP_047887999.1	CP012749.1:6060627-6062438	612.71	506.73	734.88	608.51
long-chain_fatty_acid--CoA_ligase	WP_065351161.1	CP012749.1:68329-70122	171.74	160.87	207.78	197.43

acyl--CoA_ligase	WP_082654913.1	CP015203.1:326189-327937	902.12	247.01	0.00	0.00
long-chain_fatty_acid--CoA_ligase	WP_065352992.1	CP015203.1:397563-399350	5.63	5.29	70.40	167.64
**fatty_acid--CoA_ligase**	**WP_003942422.1**	**CP015203.1:17256-18887**	**3195.26**	**361.33**	**0.17**	**0.00**
long-chain-fatty-acid--CoA_ligase	WP_058228033.1	CP015203.1:613253-614893	14.91	20.54	0.00	0.00
acyl--CoA_ligase	WP_082654938.1	CP015203.1:622905-624605	12.28	15.13	0.00	0.00
long-chain_fatty_acid--CoA_ligase	WP_081558941.1	CP015203.1:80639-82324	66.13	91.47	0.00	0.00
NAD(P)-dependent_alcohol_dehydrogenase	WP_065351586.1	CP012749.1:1835054-1836094	0.17	1.23	0.48	0.39
NAD(P)-dependent_alcohol_dehydrogenase	WP_065352366.1	CP012749.1:4869095-4870141	449.80	151.22	136.50	225.11
NDMA-dependent_alcohol_dehydrogenase	WP_003941638.1	CP012749.1:6165061-6166188	39.49	263.88	11247.61	243.28
NAD(P)-dependent_alcohol_dehydrogenase	WP_058227895.1	CP015203.1:349034-350128	10.30	83.76	0.00	0.00
NDMA-dependent_alcohol_dehydrogenase	WP_058227905.1	CP015203.1:361908-363032	39.10	290.98	0.00	0.00
alcohol dehydrogenase, propanol-preferring	WP_058228057.1	CP015203.1:649735-650709	1.92	3.35	0.00	0.00
NDMA-dependent_alcohol_dehydrogenase	WP_042920077.1	CP015203.1:66802-67908	72.74	62.94	0.00	0.00
**pyridine_nucleotide-disulfide_oxidoreductase**	**WP_003942429.1**	**CP015203.1:10252-11454**	**5187.26**	**32.13**	**0.00**	**0.19**
**pyridine_nucleotide-disulfide_oxidoreductase**	**WP_042926002.1**	**CP015203.1:53508-54716**	**13145.53**	**288.35**	**0.17**	**0.82**
rubredoxin_reductase	WP_007734096.1	CP012749.1:3106032-3107288	9037.16	18.42	7926.87	11.88

Table S3: Go Terms Enrichment of proteins expressed significantly higher in strain sp. 008 compared to strain KSM-B-3M, as identified by proteomic analysis. Four KEGG pathways (“Fatty acid degradation”, “Valine, leucine and isoleucine degradation”, “Fatty acid metabolism”, “Propanoate metabolism”) and one COG ontology (“lipid metabolism”) were highlighted, based on fourteen genes, most of them common among the pathways.

**Term**	**Benjamini**	**Genes**
Fatty acid degradation	2.57E-04	acetyl-CoA acetyltransferase (RER_RS01750)

		acetyl-CoA acetyltransferase (RER_RS01720)
		acetyl-CoA acetyltransferase (RER_RS30210)
		acyl-CoA dehydrogenase (RER_RS28070)
		alcohol dehydrogenase (RER_RS01200)
		hypothetical protein (RER_RS04060)
		acyl-CoA dehydrogenase (RER_RS01735)
		fatty-acid--CoA ligase (RER_RS02370)
		enoyl-CoA hydratase (RER_RS30095)
		acetyl-CoA acyltransferase (RER_RS28150)
Valine, leucine and isoleucine degradation	3.24E-04	acetyl-CoA acetyltransferase (RER_RS01750)
		acetyl-CoA acetyltransferase (RER_RS01720)
		acetyl-CoA acetyltransferase (RER_RS30210)
		acyl-CoA dehydrogenase (RER_RS28070)
		succinyl-CoA--3-ketoacid CoA-transferase subunit beta (RER_RS07970)
		hypothetical protein (RER_RS04060)
		dihydrolipoamide dehydrogenase (RER_RS25075)
		acyl-CoA dehydrogenase (RER_RS01735)
		enoyl-CoA hydratase (RER_RS30095)
		acetyl-CoA acyltransferase (RER_RS28150)
Fatty acid metabolism	1.25E-03	acetyl-CoA acetyltransferase (RER_RS01750)
		acetyl-CoA acetyltransferase (RER_RS01720)
		acetyl-CoA acetyltransferase (RER_RS30210)
		acyl-CoA dehydrogenase (RER_RS28070)
		hypothetical protein (RER_RS04060)
		acyl-CoA dehydrogenase (RER_RS01735)
		fatty-acid--CoA ligase (RER_RS02370)
		enoyl-CoA hydratase (RER_RS30095)
		acetyl-CoA acyltransferase (RER_RS28150)
Propanoate metabolism	1.79E-02	acetyl-CoA acetyltransferase (RER_RS01750)
		acetyl-CoA acetyltransferase (RER_RS01720)
		acyl-CoA dehydrogenase (RER_RS28070)
		hypothetical protein (RER_RS04060)
		acyl-CoA dehydrogenase (RER_RS01735)
		enoyl-CoA hydratase (RER_RS30095)
		acetyl-CoA acyltransferase (RER_RS28150)
Lipid metabolism	4.11E-02	acyl-CoA dehydrogenase (RER_RS28070)
		acyl-CoA carboxylase (RER_RS02570)
		stearoyl-CoA 9-desaturase (RER_RS12830)
		hypothetical protein (RER_RS04060)
		acyl-CoA dehydrogenase (RER_RS01735)
		fatty-acid--CoA ligase (RER_RS02370)
		enoyl-CoA hydratase (RER_RS30095)

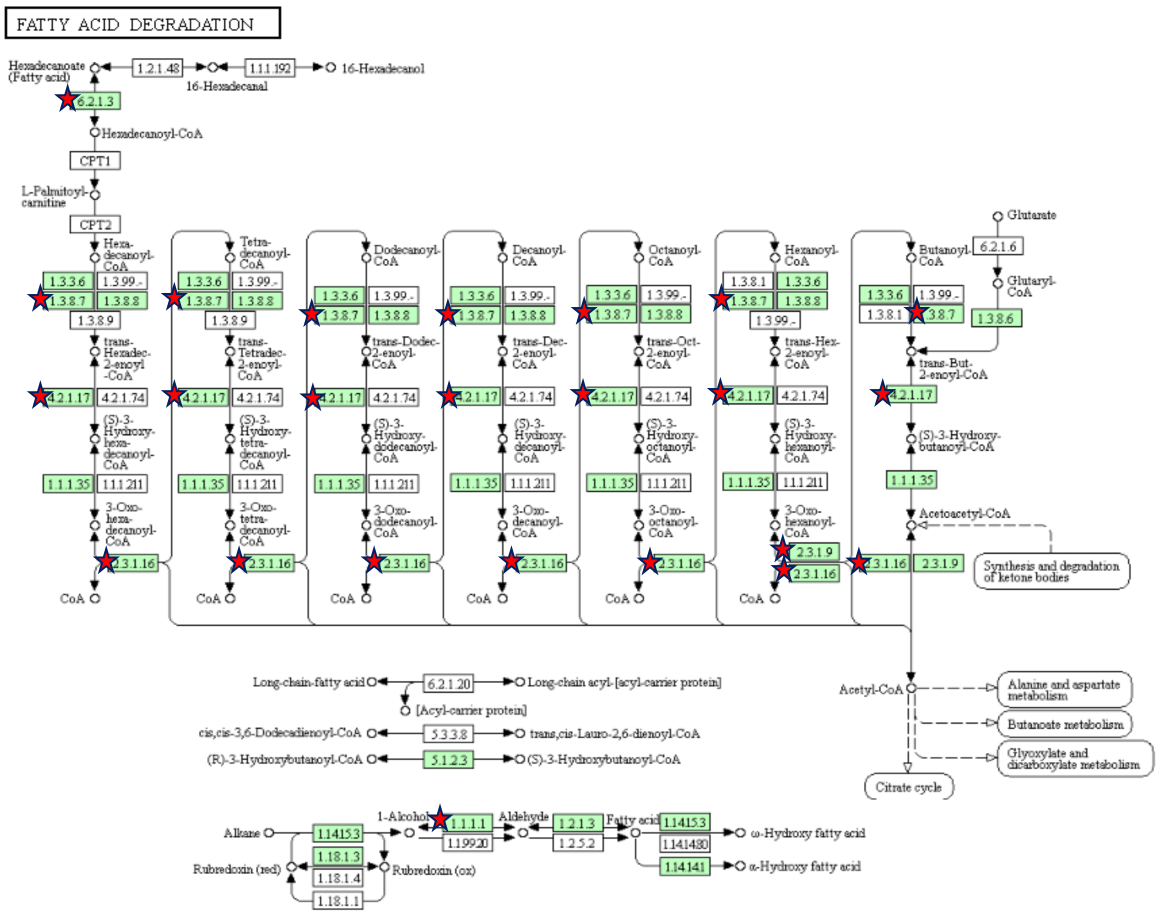

Figure S2: Down-regulation of the fatty acid degradation pathway in strain KSM-B-3M as observed by the DAVID database based on a KEGG pathway map. Red stars represent proteins detected at significantly lower abundance in the KSM-B-3M proteome compared to sp. 008.

Table S4: List of genes cured from plasmid pR8L1 (CP015203.1) of clones sp_pKO3-5 (derivates of strain sp.008). Deletion boundaries determined by Oxford Nanopore DNA Sequencing are CP015203.1:1-127890.

seqnames	start	end	Name	product
NZ_CP015203.1	233	850	WP_003946082.1	hypothetical protein
NZ_CP015203.1	993	1160	WP_003946089.1	hypothetical protein
NZ_CP015203.1	1256	1855	WP_003946084.1	hypothetical protein
NZ_CP015203.1	2087	3118	WP_003946088.1	magnesium and cobalt transport protein CorA

NZ_CP015203.1	3225	3506	WP_003946081.1	hypothetical protein
NZ_CP015203.1	3512	3712	WP_003946083.1	three-helix bundle dimerization domain-containing protein
NZ_CP015203.1	3790	4026	WP_003946087.1	hypothetical protein
NZ_CP015203.1	4118	4639	WP_231915899.1	PaaI family thioesterase
NZ_CP015203.1	4557	5153	WP_003946090.1	universal stress protein
NZ_CP015203.1	5167	6690	WP_019750069.1	MFS transporter
NZ_CP015203.1	7393	8394	WP_003942428.1	AraC family transcriptional regulator
NZ_CP015203.1	8522	8842	WP_003942421.1	2Fe-2S iron-sulfur cluster-binding protein
NZ_CP015203.1	8867	10255	WP_003942426.1	cytochrome P450
NZ_CP015203.1	10252	11454	WP_003942429.1	NAD(P)/FAD-dependent oxidoreductase
NZ_CP015203.1	11522	12985	WP_019750067.1	cytochrome P450
NZ_CP015203.1	12982	13707	WP_003942420.1	transglutaminase-like domain-containing protein
NZ_CP015203.1	13704	15233	WP_003942430.1	aldehyde dehydrogenase family protein
NZ_CP015203.1	15230	16066	WP_003942425.1	SDR family oxidoreductase
NZ_CP015203.1	16105	17172	WP_003942427.1	BtrH N-terminal domain-containing protein
NZ_CP015203.1	17256	18887	WP_003942422.1	fatty acid--CoA ligase
NZ_CP015203.1	19161	19694	WP_003942418.1	PaaI family thioesterase
NZ_CP015203.1	19691	20905	WP_003942419.1	cytochrome P450
NZ_CP015203.1	21174	21449	-	MFS transporter
NZ_CP015203.1	21578	22264	WP_042926433.1	TetR/AcrR family transcriptional regulator
NZ_CP015203.1	22892	23883		IS3 family transposase
NZ_CP015203.1	23883	25280	WP_051649791.1	IS30 family transposase
NZ_CP015203.1	25285	25599	-	integrase core domain-containing protein
NZ_CP015203.1	25869	26546	WP_167349920.1	GntR family transcriptional regulator
NZ_CP015203.1	26605	27231	WP_042926436.1	peroxiredoxin
NZ_CP015203.1	27347	27757	WP_308216554.1	class I SAM-dependent methyltransferase
NZ_CP015203.1	27732	27965	WP_306670234.1	hypothetical protein
NZ_CP015203.1	27962	28309	WP_042925967.1	DsrE family protein

NZ_CP015203.1	28347	29219	WP_042925969.1	cytochrome c biogenesis CcdA family protein
NZ_CP015203.1	29231	29959	WP_042925970.1	DsbA family protein
NZ_CP015203.1	30066	30578	WP_042925971.1	hypothetical protein
NZ_CP015203.1	30654	31061	WP_042925972.1	DUF302 domain-containing protein
NZ_CP015203.1	31115	32233	WP_231917489.1	hypothetical protein
NZ_CP015203.1	32256	32471	WP_006898201.1	YgaP-like transmembrane domain
NZ_CP015203.1	32505	33380	WP_074447159.1	sigma-70 family RNA polymerase sigma factor
NZ_CP015203.1	34014	34487	WP_058228376.1	hypothetical protein
NZ_CP015203.1	34484	36257	-	aa3-type cytochrome oxidase subunit I
NZ_CP015203.1	36337	37179	WP_065352978.1	sulfite exporter TauE/SafE family protein
NZ_CP015203.1	37181	37519	WP_042925975.1	rhodanese-like domain-containing protein
NZ_CP015203.1	37548	38780	WP_231915860.1	GntR family transcriptional regulator
NZ_CP015203.1	38808	40202	WP_042925976.1	MBL fold metallo-hydrolase
NZ_CP015203.1	40781	41986	WP_054186660.1	NAD(P)/FAD-dependent oxidoreductase
NZ_CP015203.1	41983	42519	WP_042925977.1	Rv2732c family membrane protein
NZ_CP015203.1	42613	42876	WP_042925979.1	metal-sensitive transcriptional regulator
NZ_CP015203.1	42992	45172	WP_054186659.1	MMPL family transporter
NZ_CP015203.1	45307	45552	WP_042926020.1	YgaP family membrane protein
NZ_CP015203.1	45549	45980	WP_042925988.1	DUF5313 family protein
NZ_CP015203.1	45984	46619	WP_042925990.1	class I SAM-dependent methyltransferase
NZ_CP015203.1	46954	47658	WP_042925992.1	TetR/AcrR family transcriptional regulator
NZ_CP015203.1	47862	49337	WP_372510032.1	MFS transporter
NZ_CP015203.1	49354	49908	WP_042925998.1	PaaI family thioesterase
NZ_CP015203.1	50355	51419	WP_042926000.1	BtrH N-terminal domain-containing protein
NZ_CP015203.1	51466	52302	WP_054186658.1	SDR family oxidoreductase

NZ_CP015203.1	52698	53438	WP_054186661.1	transglutaminase-like domain-containing protein
NZ_CP015203.1	53508	54716	WP_042926002.1	NAD(P)/FAD-dependent oxidoreductase
NZ_CP015203.1	54713	56101	WP_042926022.1	cytochrome P450
NZ_CP015203.1	56126	56446	WP_042926005.1	2Fe-2S iron-sulfur cluster-binding protein
NZ_CP015203.1	56673	58424	WP_042926008.1	FAD-dependent oxidoreductase
NZ_CP015203.1	58855	60108	WP_042926010.1	PucR family transcriptional regulator
NZ_CP015203.1	60162	60566	WP_128638070.1	hypothetical protein
NZ_CP015203.1	61278	62582	WP_042927240.1	IS256-like element ISRer3 family transposase
NZ_CP015203.1	62710	63363	WP_042920075.1	TetR family transcriptional regulator
NZ_CP015203.1	63563	64591	WP_051649474.1	helix-turn-helix domain-containing protein
NZ_CP015203.1	64724	65842	WP_051649475.1	hypothetical protein
NZ_CP015203.1	65839	66096		hypothetical protein
NZ_CP015203.1	66802	67908	WP_042920077.1	NDMA-dependent alcohol dehydrogenase
NZ_CP015203.1	67937	68947	WP_042920079.1	NAD(P)/FAD-dependent oxidoreductase
NZ_CP015203.1	68975	69292	WP_042920082.1	ferredoxin
NZ_CP015203.1	69382	70377	WP_042920084.1	R2-like ligand-binding oxidase
NZ_CP015203.1	71381	71731	WP_074447157.1	transposase family protein
NZ_CP015203.1	71664	72683	WP_081558937.1	transposase
NZ_CP015203.1	72711	73142	WP_051649477.1	tyrosine-type recombinase/integrase
NZ_CP015203.1	73139	73411	WP_058228369.1	hypothetical protein
NZ_CP015203.1	73590	74102	-	ATP-binding protein
NZ_CP015203.1	74175	75426	-	Mu transposase domain-containing protein
NZ_CP015203.1	75494	75922	-	IS1380 family transposase
NZ_CP015203.1	76002	76688	WP_058228370.1	response regulator transcription factor
NZ_CP015203.1	76791	78125	WP_051649478.1	sensor histidine kinase

NZ_CP015203.1	78428	78583	WP_156519336.1	hypothetical protein
NZ_CP015203.1	78646	80130	WP_042920093.1	aldehyde dehydrogenase family protein
NZ_CP015203.1	80143	80553	WP_042920096.1	sterol carrier protein
NZ_CP015203.1	80639	82324	WP_081558941.1	long-chain fatty acid--CoA ligase
NZ_CP015203.1	82700	83377	WP_231915856.1	MlaE family ABC transporter permease
NZ_CP015203.1	83519	84298	WP_081559622.1	MlaE family ABC transporter permease
NZ_CP015203.1	84300	85463	WP_058228372.1	MCE family protein
NZ_CP015203.1	85460	86464	WP_042920102.1	MCE family protein
NZ_CP015203.1	86464	87462	WP_042920103.1	MCE family protein
NZ_CP015203.1	87459	88565	WP_042920106.1	MCE family protein
NZ_CP015203.1	88562	89701	WP_042920109.1	MCE family protein
NZ_CP015203.1	89701	91161	WP_042920112.1	MCE family protein
NZ_CP015203.1	91137	91736	WP_042920115.1	hypothetical protein
NZ_CP015203.1	91733	92263	WP_042920118.1	hypothetical protein
NZ_CP015203.1	92323	93375	WP_042920120.1	ABC transporter ATP-binding protein
NZ_CP015203.1	93791	93973	WP_128637843.1	hypothetical protein
NZ_CP015203.1	95045	95236	WP_042920127.1	hypothetical protein
NZ_CP015203.1	95354	95662	WP_058228373.1	hypothetical protein
NZ_CP015203.1	95837	97741	WP_042920323.1	alkyl/aryl-sulfatase
NZ_CP015203.1	97894	98481	WP_058228374.1	DUF4203 domain-containing protein
NZ_CP015203.1	98815	99390	-	steroid 3-ketoacyl-CoA thiolase
NZ_CP015203.1	99697	100062	WP_019747233.1	ArsR/SmtB family transcription factor
NZ_CP015203.1	100185	101042	WP_042920135.1	hypothetical protein
NZ_CP015203.1	101111	103009	WP_042920139.1	phosphoenolpyruvate carboxykinase (GTP)
NZ_CP015203.1	103164	103337	WP_154606736.1	hypothetical protein
NZ_CP015203.1	103424	105154	WP_042920142.1	SulP family inorganic anion transporter
NZ_CP015203.1	105161	105547	WP_042920144.1	DUF5313 family protein
NZ_CP015203.1	105544	105672	WP_257784574.1	hypothetical protein
NZ_CP015203.1	105766	106962	WP_042920147.1	acetyl-CoA C-acetyltransferase

NZ_CP015203.1	107129	107476	WP_042926495.1	zinc ribbon domain-containing protein YjdM
NZ_CP015203.1	107463	107984	WP_042926483.1	hypothetical protein
NZ_CP015203.1	108174	109319	WP_042926492.1	thiolase family protein
NZ_CP015203.1	109543	109830	WP_042926481.1	meromycolate extension acyl carrier protein AcpM
NZ_CP015203.1	109827	111083	WP_042926479.1	KasA/KasB family beta-ketoacyl-ACP synthase
NZ_CP015203.1	111107	111928	WP_042926476.1	alpha/beta fold hydrolase
NZ_CP015203.1	111925	112878	WP_042926474.1	hypothetical protein
NZ_CP015203.1	113072	113230	WP_167349918.1	hypothetical protein
NZ_CP015203.1	113734	113874	WP_231917508.1	hypothetical protein
NZ_CP015203.1	114097	114767		transposase
NZ_CP015203.1	114970	115041	WP_408990032.1	helix-turn-helix domain-containing protein
NZ_CP015203.1	115082	116707	WP_231917490.1	hydantoinase/oxoprolinase family protein
NZ_CP015203.1	116773	117209	-	acetone carboxylase subunit gamma
NZ_CP015203.1	117232	119358	WP_065352980.1	hydantoinase/oxoprolinase family protein
NZ_CP015203.1	119408	121735	WP_042926467.1	hydantoinase B/oxoprolinase family protein
NZ_CP015203.1	121834	122643	WP_042926486.1	hypothetical protein
NZ_CP015203.1	123291	123836	WP_328587284.1	DDE-type integrase/transposase/recombinase
NZ_CP015203.1	123752	124303	-	PDDEXK nuclease domain-containing protein
NZ_CP015203.1	124432	125229	WP_058228359.1	hypothetical protein
NZ_CP015203.1	125229	127190	WP_231917492.1	hypothetical protein
NZ_CP015203.1	127207	127521	WP_042926284.1	type II toxin-antitoxin system PemK/MazF family toxin
NZ_CP015203.1	127522	127743	WP_042926289.1	hypothetical protein

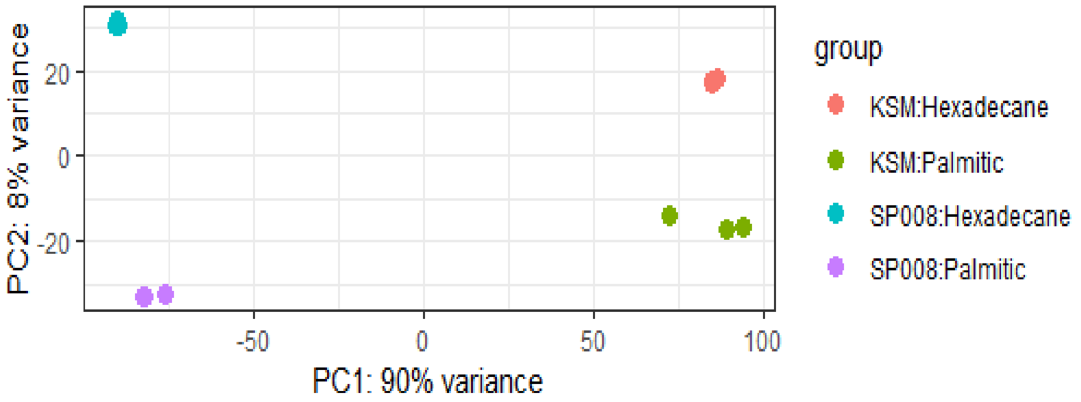

Figure S3*:* Principal component analysis (PCA) demonstrating the transcriptomic profiles of strains KSM-B-3M and sp. 008 grown on *n*-hexadecane or palmitic acid. Pink, strain KSM-B-3M grown on n-hexadecane; green, strain KSM-B-3M grown on palmitic acid; blue, strain sp. 008 grown on n-hexadecane; purple, strain sp. 008 grown on palmitic acid. The transcriptome of each strain and condition was tested in triplicates. 90% of the variation is explained by PC1 which corresponds to differences between strains.

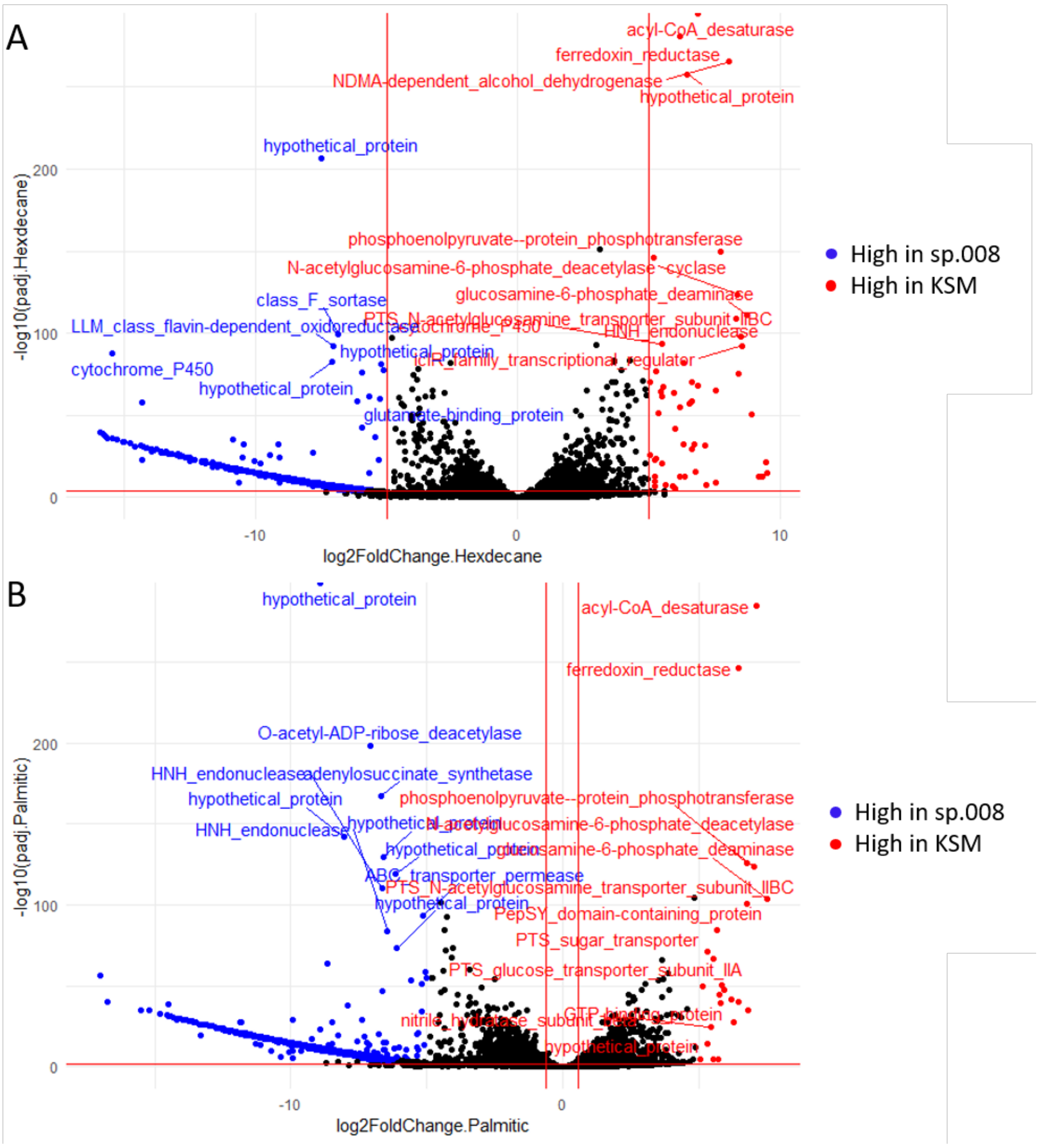

Figure S4: Differential gene expression between strains KSM-B-3M and sp. 008. Volcano plots represent the significance of each gene expression vs. its expression differential. A. Strains were grown on *n*-hexadecane (replication to the experiment presented in Figure 3); B. Strains were grown on palmitic acid; Red, excessive in strain KSM-B-3M by 2^5 fold change; blue, excessive in strain sp. 008 by 2^{10} fold change. The acyl-CoA desaturase was the most highly expressed gene in strain KSM-B-3M under either *n*-hexadecane or palmitic acid.

Table S5: Substrates divided according to their dehydrogenation productivity by strain KSM-B-3M. Substrates demonstrating "High productivity" and "low productivity" corresponds to conversion ratios higher or lower than 15%, respectively; "No productivity" substrates demonstrated no detectable conversion. Conversion ratios were measured after 10 incubation days of strain KSM-B-3M in medium A containing the different substrates.

**High productivity**	**Low productivity**	**No productivity**
n-hexadecane (C16)	*n*-dodecane (C12)	N,N-Dimethylhexadecylamine (C18)
n-octadecane (C18)	*n*-undecane (C11)	Hexadecanal (C16)
n-heptadecane (C17)	2-Methyl-octadecane (C19)	1-Iodohexadecane (C16)
1-Chlorohexadecane (C16)	1-butoxytetradecane (C16)	Tetradecylcyclopropane (C17)
1-Methoxyhexadecane (C17)	2-Pentadecyl-1,3-dioxalane (C18)	1-(hexyloxy)decane (C16)
n-pentadecane (C15)	1-Azidohexadecane (C16)	1,2-dichlorohexadecne (C16)
1-Flourohecadacane (C16)		*cis*-1-hexyl-2-octycyclopropane (C17)
n-tetradecane (C14)		Heptadec-1-yne (C17)
1-Hexadecene (C16)		Hexadecyl(methyl)sulfane (C17)
		(Hexadecycloxy)trimethylsilane(C19)

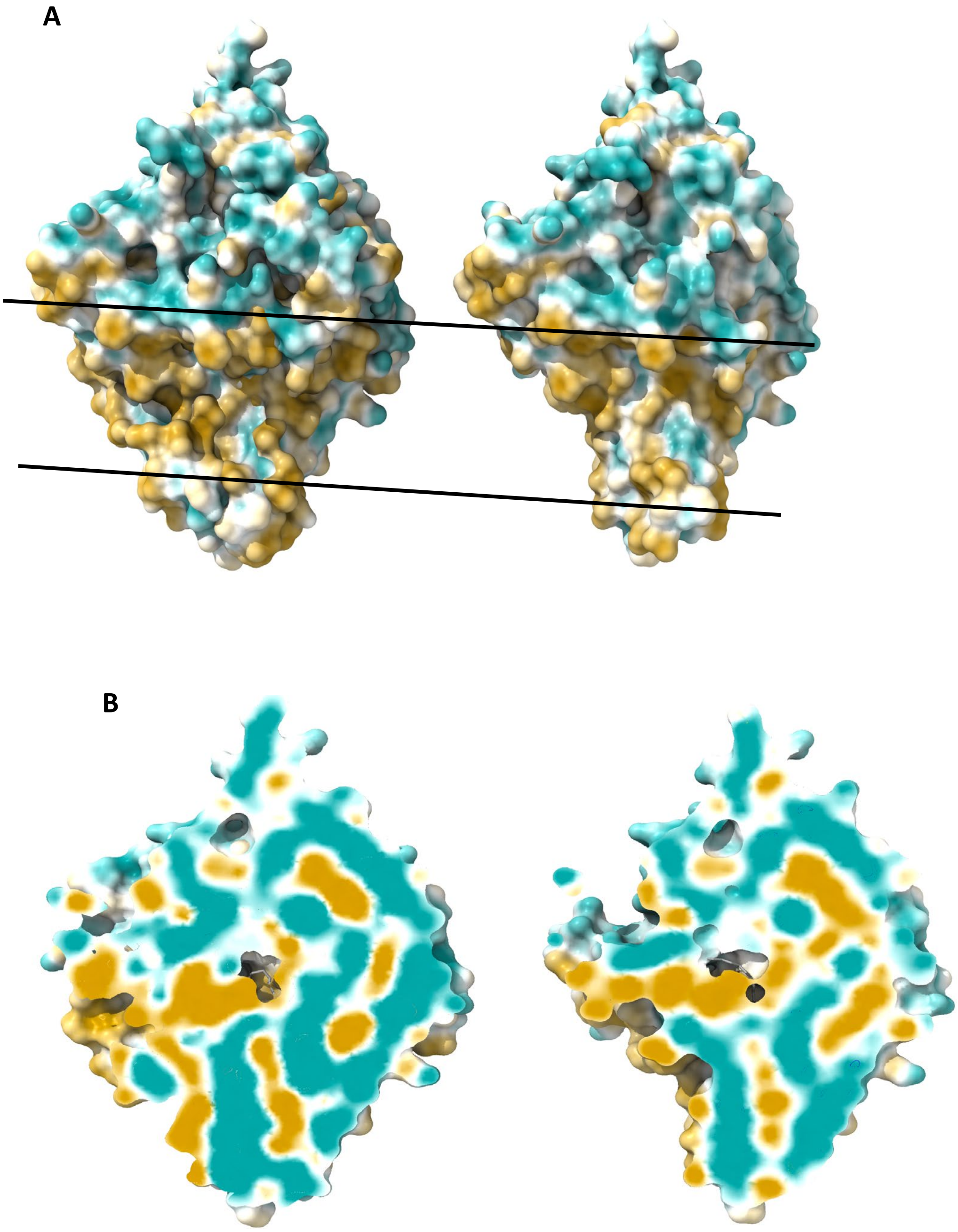

Figure S5: The hydrophobicity pattern of Mouse and *Rhodococcus* desaturases. A. Mouse structure and *Rhodococcus* model surface representation and colored coded by hydrophobicity, approximate trans-membrane edges are indicated by black lines. B. Hydrophobicity pattern at the clipping plane crossing the binding sites of both molecules, where the substrates are partially visible.

Variation in expression levels of the operon genes under alkanes and fatty acids.

QPCR was utilized to examine the operon expression patterns under *n*-hexadecane and palmitic acid. When cultivated on *n*-hexadecane, the expression levels of all three operon genes were notably higher in strain KSM-B-3M compared to strain sp. 008, consistent with the transcriptomic analyses (Figures 3, S3); levels of acyl CoA desaturase, ferredoxin reductase, and the hypothetical protein measured in strain KSM-B-3M were elevated by 70, 80 and 40-fold, respectively, compared to strain sp. 008 (Figure S4A). Interestingly, the expression levels of all three genes in strain KSM-B-3M were 5 to 10-fold higher when grown on *n*-hexadecane compared to palmitic acid (Figure S4A). A similar trend, albeit with significantly lower expression levels, was observed for strain sp. 008 (Figure S4B). This suggests that along with the inherently higher operon expression observed for strain KSM-B-3M, the intensified operon expression under *n*-hexadecane compared to palmitic acid represents a general phenomenon rather than being strain-specific. To further explore the phenomenon of increased operon expression under alkanes versus fatty acids, further assays were conducted. The expression levels of acyl-CoA desaturase in strain KSM-B-3M were examined across a range of alkanes (*n*-hexadecane, *n*-dodecane and *n*-heptadecane) and fatty acids (palmitic and stearic acids); consistently, acyl-CoA desaturase expression levels were significantly higher when grown on alkanes compared to saturated fatty acids (Figure S5; $p < 0.001$). Indeed, palmitic and stearic acids serve as the natural substrates for acyl-CoA desaturase, with their dehydrogenation products - palmitoleic and oleic acid - acting as negative signaling molecules for acyl-CoA desaturase[1]. This

may partially elucidate the elevated and probably unregulated levels of acyl CoA desaturase when alkanes are utilized as reaction substrates, as opposed to fatty acids.

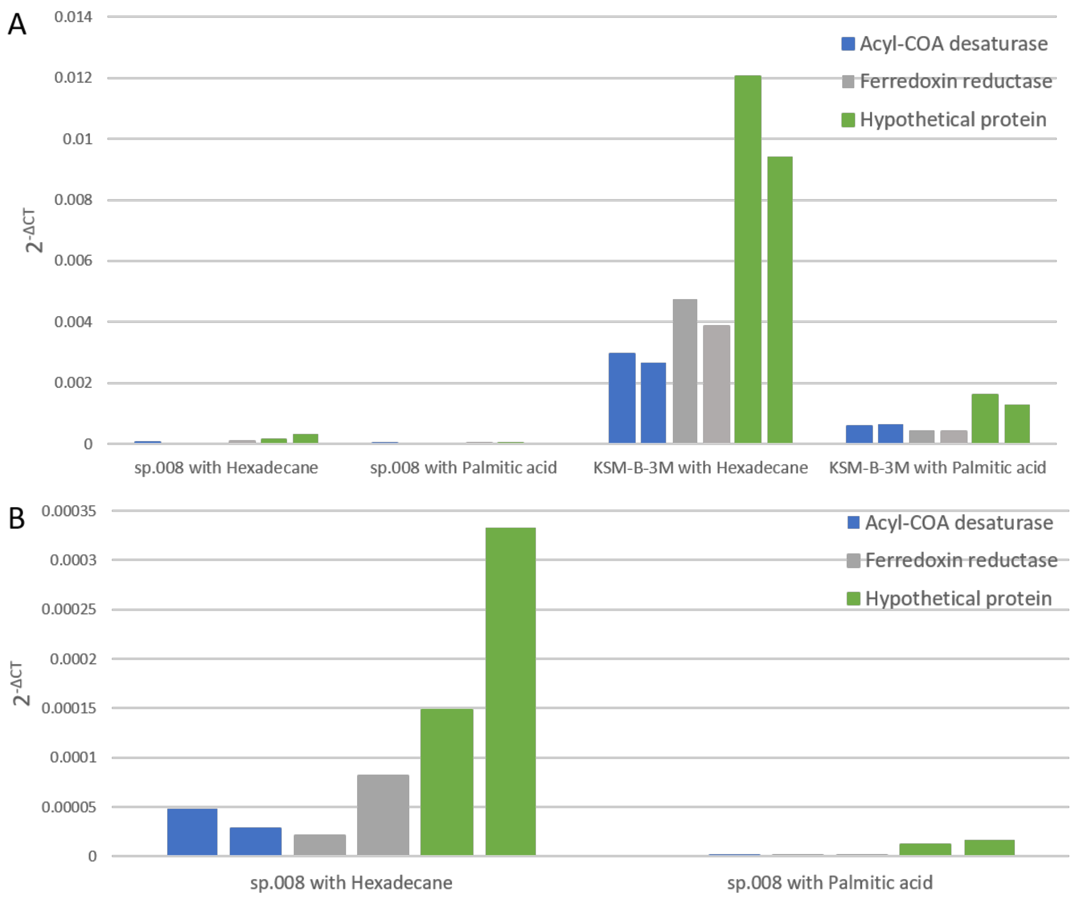

Figure S6: Expression levels of acyl-CoA desaturase, ferredoxin reductase and the gene encodes for hypothetical protein as obtained on *n*-hexadecane and palmitic acid. A. Expression levels obtained for both sp. 008 and KSM-B-3M strains. B. Enlargement of the expression levels obtained for strain sp. 008 demonstrated in (A). Q-PCR was used to assess expression levels after 7 incubation days on medium A supplemented with *n*-hexadecane or palmitic acid, with two biological repeats for each treatment. Expression levels of the analyzed genes were calculated by comparative C_T method ($2^{-\Delta CT}$) with both 23s rDNA and 16s rDNA used as the reference genes.

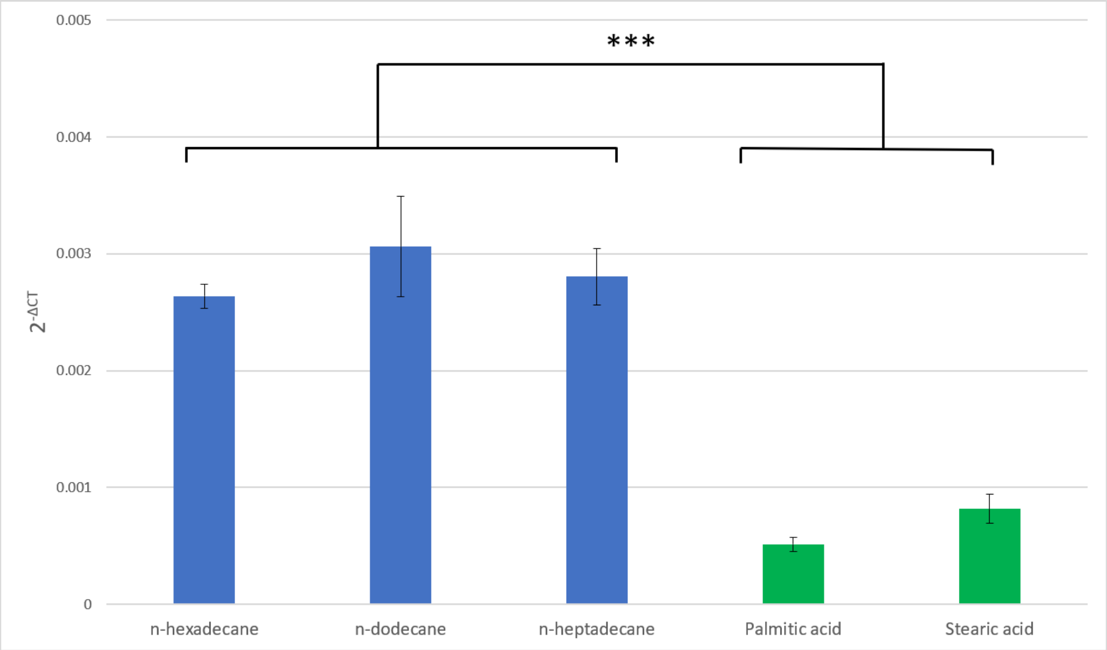

Figure S7*:* Acyl-CoA desaturase expression in strain KSM-B-3M grown on alkanes (blue bars) vs. saturated fatty acids (green bars) as determined by qPCR analysis. Expression levels were calculated using comparative C_T method ($2^{-\Delta CT}$) with 23s rDNA and 16s rDNA used as the reference genes. Values are averages of biological triplicates for each treatment. ****, p < 0.001*, comparing alkanes to fatty acids.

1. Aguilar, P.S.; De Mendoza, D. Control of Fatty Acid Desaturation: A Mechanism Conserved from Bacteria to Humans. *Molecular Microbiology* **2006**, *62*, 1507–1514, doi:10.1111/j.1365-2958.2006.05484.x.
